# Supplementary material for: Acceptability of test and treat with doxycycline against Onchocerciasis in an area of persistent transmission in Massangam Health District, Cameroon
Source: PLoS Negl Trop Dis. 2023 Apr 5;17(4):e0011185. doi: 10.1371/journal.pntd.0011185 (PMC10075443; doi:10.1371/journal.pntd.0011185)
Supplement: S1 Text — (PDF) [file pntd.0011185.s001.pdf]

REPUBLIQUE DU CAMEROUN
Paix- Travail- Patrie
-----------
MINISTERE DE LA SANTE PUBLIQUE
---------------
SECRETARIAT GENERAL
-----------------
DIRECTION DE LA LUTTE CONTRE LA MALADIE, LES EPIDEMIES ET LES PANDEMIES
-----------
SOUS- DIRECTION EN CHARGE DE LA LUTTE CONTRE LE PALUDISME ET LES MALADIES TROPICALES NEGLIGEES

REPUBLIC OF CAMEROON
Peace-Work-Fatherland
---------
MINISTRY OF PUBLIC HEALTH
-----------
SECRETARIAT GENERAL
-----------
DEPARTMENT OF DISEASES CONTROL, EPIDEMIC AND PANDEMIC
-----------
SUB-DEPARTMENT IN CHARGE OF MALARIA AND TROPICAL NEGLETED DISEASES

# RAPPORT DE LA REUNION DES INTERVENANTS SUR LA RECHERCHE DES STRATEGIES ALTERNATIVES DANS LA LUTTE CONTRE L'ONCHOCERCOSE

*07 et 08 février 2017*

*A la salle de conférences de la Direction de la lutte contre la Maladie, les Epidémies et les Pandémies*

Les 07 et 08 février 2017, s'est tenue dans la salle de réunion de la DLMEP une réunion des intervenants sur la recherche des stratégies alternatives dans la lutte contre l'Onchocercose présidée par Monsieur le SDLPMTN en lieu et place de Monsieur le DLMEP empêché.

Y ont pris par à cette réunion outre les responsables du Minsanté (CS/MTN, SP/ONCHO, SPA/ONCHO, DRSP/OUEST, PF/MTN/OUEST, Rep DROS, CSSD/Massagam, Cadre SDLPMTN), les partenaires ci après :

- Dr Oye Joseph, Country Director Sightsavers-Cameroun,
- M. Akongo Serge, PM NTDs (Sightsavers-Cameroun),
- Dr Didier Bakajika, Epidemiologiste (Sightsavers),
- Dr Fobi Gracen consultant SightSavers,
- Mme Akame Julie, Programme Manager / HKI,
- M. Moungui Henry, M&E Officer/HKI,
- Prof Samuel Wanji, Parasitologue (Université de Buea),
- Dr Peter Enyong, Entomologiste (Université de Buea),
- M. Nkouelle Alexis, IEF représentative.

Cette réunion a pour objectif la validation du document de recherche opérationnelle pilote élaboré pour implémenter les stratégies alternatives dans le cadre de la lutte contre l'Onchocercose, dans le District de Santé de Massagam dans la Région de l'Ouest Cameroun.

## I- CONTEXTE

Les résultats obtenus à la suite des enquêtes épidémiologiques conduites dans la Région de l'Ouest Cameroun en 2011 et 2015, et ceux des évaluations entomologiques ayant pour but de déterminer le potentiel d'infestivité des simulies conduites en 2016, révèlent que la transmission de l'onchocercose se poursuit dans l'Aire de santé de Massagam situé le District de sante de Massagam, malgré des taux de couverture thérapeutique annuels soutenus supérieurs à 80%. D'où la nécessité de développer des stratégies alternatives.

REPUBLIQUE DU CAMEROUN Ministère de la Santé Publique Le Chef d'Unité

## II- PRESENTATION DU DOCUMENT DE RECHERCHE ET DES STRATEGIES ALTERNATIVES A IMPLEMENTER

le Prof Wanji de l'Université de Buea a présenté le document de recherche élaboré à la suite des recommandations de la réunion du comité d'expert de mai 2016, tenue à l'hôtel Mont Fébé à Yaoundé, sous la coordination du Ministère de la sante publique.

Il s'agira de réaliser une étude pilote afin d'évaluer l'efficacité de ces stratégies alternatives.

Le présent document résume les stratégies à implémenter dans la zone cible, à savoir :

- **l'administration de la doxycycline** dans 5 communautés de l'aire de sante de massagam (par la stratégie de « Test and Treat » :

La distribution de la doxycycline s'appuiera sur le système de santé, avec formation en cascade qui ira du district sanitaire jusqu'aux relais communautaires.

Toutes les personnes vivant dans les cinq communautés "hotspot" de la zone de Massagam, âgées de 10 ans et plus, seront testées pour l'onchocercose par la méthode de biopsie cutanée exsangue. Ces personnes seront aussi testées pour la loase par la méthode de goutte épaisse calibrée.

Les personnes porteuses de microfilaires d'*O. volvulus* seront traitées à la Doxycycline si éligible au traitement (test de grossesse pour les femmes négative, etc.) :

La doxycycline sera obtenue par le truchement de Liverpool School of Tropical Médicine.

- **la distribution biannuelle du mectizan** dans les autres aires de sante du District,

Les personnes ne portant pas les microfilaires de d'*O. volvulus* prendront le Mectizan si elles ne sont pas porteuses de microfilaire de *Loa loa au seuil de danger défini dans le Guide MEC/TCC.* Le PNLO dispose d'un stock the Mectizan pour ce travail.

- **la lutte anti vectorielle** ciblée utilisant des larvicides sélectionnés, dans 8 sites de transmission. Le Themephos sera utilisé dans le cadre de ce projet et sera obtenu par le truchement d'un fournisseur local basé à Douala

A la suite de cette présentation des discussions ont permis d'éclaircir certains points des stratégies à implémenter :

### 1- Plan de communication pour le projet

Un grand accent sera mis sur la communication et la sensibilisation tout en tenant compte de contexte socio-politique de la zone de Massagam. Il s'agit notamment de conduire les activités suivantes :

- Réunion de plaidoyer avec les élus locaux, les autorités administratives
- Lancement officiel de la stratégie
- Réunion avec les différents chefs de village
- sensibilisation de membres des communautés
- réunion avec les structures de dialogue.

### 2- Institution du comité de suivi

Un comité de suivi du projet sera mis en place et aura une dizaine de membres:

- ✓ Le Maire de Massagam
- ✓ Le députe de Massagam
- ✓ le chef de groupement

REPUBLIQUE DU CAMEROUN MINISTERE DE LA SANTE PUBLIQUE Le Chef d'Unité

- ✓ DLMEP Représentant
- ✓ PNLO
- ✓ DROS
- ✓ DRSP/ROC
- ✓ DPML/UCNLMTN
- ✓ ONG partenaire
- ✓ L'équipe scientifique
- ✓ Délègue Régional en charge des questions de l'environnement

### 3- Site Temoin, sans Test and Treat

Il a été propose à l'équipe des chercheurs de trouver une zone plus au sud de Massagam y compris dans le DS de Bafia, qui pourrait servir de site temoin. Une évaluation épidémiologique pourra y être conduite. Ce deuxième site d'étude aura pour seule intervention additive la lutte anti-vectorielle.

## III- RECOMMANDATION

A l'issu des échanges des recommandations à mettre en œuvre avant le démarrage du projet ont été formulés :

| N | Recommandations | Responsables |
|---|---|---|
| 1 | - Mettre en place un mécanisme de motivation des relais communautaires engagés dans le projet. | Equipe de Recherche<br>ONG Partenaire |
| 2 | - Définir clairement dans le projet de recherche un cadre opérationnel de supervision et de suivi des activités | DLMEP<br>ONG partenaire |
| 3 | - Mettre en place un comité de suivi et définir son mode de fonctionnement dans le document projet | DLMEP<br>PNLO<br>ONG partenaire |
| 4 | - Déterminer un site témoin pour l'étude qui pourrait avoir avec pour seule intervention la lutte anti-vectorielle. | Equipe de recherche |
| 5 | - Prendre en compte l'évaluation cout/efficacité du projet et l'insérer dans le projet de recherche. | Equipe de recherche<br>ONG partenaire |

## IV- PROCHAINE ETAPES

| Activités | Echéance |
|---|---|
| 1- Révision du document de recherche | 15 février |
| 2- Soumission à la commission éthique | 15 Février |
| 3- Elaboration d'un plan et d'un budget de communication | 20 février |
| 4- Recruter un assistant de recherche (Sightsavers) | 15 Mars |
| 5- Elaborer les documents de formation des personnels et des relais communautaires | 15 Mars |

Fait à Yaoundé, le 10 février 2017

Président de Séance

Dr. NKO'AYISSI Georges
MD Msc MPH DU Epistat
(FMSB-UY1 ISPED-Bordeaux 2)
Médecin-Epidémiologiste

Rapporteur

REPUBLIQUE DU CAMEROUN
MINISTERE DE LA SANTE PUBLIQUE
Le Chef d'Unité
