# Supplementary material for: Acceptability of test and treat with doxycycline against Onchocerciasis in an area of persistent transmission in Massangam Health District, Cameroon
Source: PLoS Negl Trop Dis. 2023 Apr 5;17(4):e0011185. doi: 10.1371/journal.pntd.0011185 (PMC10075443; doi:10.1371/journal.pntd.0011185)
Supplement: S2 Text — (PDF) [file pntd.0011185.s002.pdf]

ACCELERATING THE ELIMINATION OF HUMAN ONCHOCERCIASIS INFECTION AND TRANSMISSION THROUGH ALTERNATIVE TREATMENT STRATEGIES IN THE MASSANGAM HEALTH DISTRICT IN WEST CAMEROON.

55RES - 12141

Report on the First Phase of Doxycycline Treatment

December 19, 2017

By:

Dr Rogers NDITANCHOU
Regional Research Advisor – NTDs
Kareen Atekem
Research Coordinator – NTD's

1. Introduction

Despite more than 15 years of Ivermectin mass drug distribution in Massangam Health District (HD), studies have revealed that there is a high prevalence of infection and transmission of *Onchocerca volvulus* is still on going. Based on current interventions (annual Ivermectin), it is highly unlikely Massangam will achieve elimination targets by 2025, and therefore a need for an alternative strategy (as compared to annual Ivermectin distribution) in order to accelerate elimination timelines. These strategies involve test and treat with doxycycline in the hotspot communities, biannual mectizan distribution in the communities surrounding the hotspot and ground larviciding.

For the Doxycycline test and treat strategy, screening was done in August 2017, two weeks following IVM distribution in the hotspot. The first phase of treatment with doxycycline which is intended to treat those screened positive, started on the 11th of December 2017 in the three-hotspot communities of Massangam Health Area.

The following activities were carried out before and during this first phase:

- Mobilisation and sensitization
- Training of CDDs
- Advocacy and Launching
- Doxycycline treatment and monitoring

1.1. Mobilization and Sensitization

Community mobilization and sensitization that started a week before was intensified from the 8th of December and extend throughout the treatment period. The aim of this activity was to mobilize local stakeholders and sensitize the community on the treatment with doxycycline. The various methods below were used during community mobilization and sensitization:

- **Community mobilisation and sensitization meeting**

The first day was consecrated to mobilisation and sensitization meeting. This involved the district team, all front line health personnel of both the hotspot and surrounding communities, focal persons for communication, community and religious leaders and CDDs involved in community drug distribution in the hotspots. During this meeting, the purpose of the Massangam Initiative was explained and the importance of community awareness and adherence to treatment emphasized.

During the question and answer session, the following points were raised:

Table 1: Points discussed during question and answer session

Questions/ Issue raised	Discussion around question
How do we know if the age of the child is 9 years?	Rely base on what the parents say, if possible ask for child's document
How do we know if a woman is pregnant when taking treatment?	Ask if the woman is pregnant, in case not visible; do urine test to confirm (at the beginning, midway and the end of treatment); counsel woman not to get pregnant or use birth control; if pregnant, woman must stop treatment at once.
How do we eliminate the disease within the hotspot when the population is mobile because of agricultural activities?	Identify these people who are very mobile; identify the periods of the year when they are present and distribute at this time; integrate the Mbororo group to identify which community has migrated to which area.
Which diseases are considered as chronic that contraindicate treatment with doxycycline?	Generally, the diseases mentioned in the checklist are not contra-indicative to doxycycline but in case they are very sick and/or taking medication for these conditions, they will not take part to avoid drugs interaction or false

	information spreading in case conditions worsen. The CDDs should inform the Chief of Centres (CoCs) and even the District Medical Officer (DMO) in case of any of the above conditions or allergy.
What do we do if a person starts treatment and wants to travel out?	Give the person his/her drug to travel with but emphasize the person eats before drinking it. And that the empty medicine blister should be returned
What do we do with persons coming into the community when they were not screened and they want to take doxycycline?	Do not give treatment but sensitize on the next round of screening and treatment.
What happens if someone was screened in one community during screening and resides in another during treatment?	- Inform the person to come and follow his/her treatment in the community where screening took place; if not possible the CDD of the community he/she is residing will follow-up and that will be a transfer case.<br>- The CDDs should work in close collaboration with the community leaders, as they are well informed of the movement of people in their communities.

Some concerns were raised to understand why the number screened was lower than the number censored. The following reasons were mentioned and some actions proposed to mitigate the problem against the next round of activities (Table 2).

Table 2: Reasons for lower coverage rate during screening

Problem	Explanation	Action proposed
**Communication**	The information about screening was sent out late and there was not enough time for the information to circulate before screening started.	Sensitize and mobilize the community ahead of time to allow for information to circulate
**Immigration**	The population is highly mobile due to their agricultural and dairy farming activities	Integrate Mbororo to work with CDDs and coincide treatment with farming period
**Refusal**	Refusal arose because results of previous screening of 2015 were not made known to the persons concerned or because of concern of their skin cut off to unknown place	Results are made available for those who want to know (negative cases) and those positive are treated
**Non-respect of time on the part of SSI**	Activities were not followed according to plan	Respect chronogram of activities

- **Communique to churches, mosques and village meetings**

Key messages in the form of communiques signed by Sub-Divisional Officer (SDO) were sent to Mosques on Fridays, to association/”Njangi”/tribal meetings of Saturday, and to churches on Sundays to inform the entire community about doxycycline treatment. In addition, these messages were transferred to village meetings, women gatherings, and other community gatherings through specific individuals present during the sensitization and mobilisation meeting.

- **BCC tools (posters, banners and flyers)**

Banners and posters were hoisted and posted (respectively) in market places, hospitals and health centres, churches, mosques, palaces, houses of religious leaders and other public places. Flyers were equally distributed to everybody during the sensitization meeting as well as during the launching ceremony.

- **One-to-one sensitization**

This was a face-to-face discussion between the CDD and patients in their respective communities prior to treatment. The CDDs further explained the result and importance of treatment and emphasized the need to take treatment for the 35 days. Any case of resistance/refusal was referred to the supervising team (REFOTDE and Sightsavers). Using the flyers distributed, they explained to their patients the burden of the disease not only to them but also to their family and the community at large in case of refusal that could lead to reinfection.

1.2. Training of health personnel and CDDs

The next two days were for training of health personnel and CDDs and alongside mobilisation and sensitization. Twenty CDDs and two CoCs from the three-hotspot communities and District team were trained. On the first day, training was more theoretical and based on pre-established training module. The following points were covered during training:

- Onchocerciasis and socio-economic impact
- Treatment of Onchocerciasis - Mectizan in surrounding communities and doxycycline in hotspot communities
- Doxycycline advantages, dosage, possible side effects and how to manage the various categories (mild, moderate and severe)
- Packaging of doxycycline per week for each patient to be followed up
- Consent and ascent forms
- Method of doxycycline administration and timing
- Filling of doxycycline treatment forms/registers and follow up of patients
- Conducting pregnancy test
- Storing and handling of registers, forms and medicine

Each CDD was given a training module and they read in turns. This allowed us to judge their reading proficiency in addition to a written test. This way we were able to identify two CDDs as unfit and limited their role to that of support. The CDDs kept their training modules to refer to when necessary.

The second day of training was in the communities and this was a one-to-one exchange between the CDD and the trainer to emphasize on key points and identify individual challenges that were not handled the previous day. It was also during this time that the CDDs identified their various food points and the person(s) who will be in charge of breakfast for

the patients. The time for distribution of doxycycline was agreed to be from 6-8am in order to capture majority of the persons before they go to their farms or start their daily activities.

1.3. Advocacy and launching

Launching of doxycycline treatment took place on the 11th of December in the presence of local stakeholders, local administrator (SDO), MoH (Central and Regional level), District team, CoCs of both hotspot and surrounding communities, religious leaders, political leaders, CDDs, REFOTDE and Sightsavers. Several speeches were made including that of the SDO, the Regional Delegate of Public Health, the Mayor of Massangam, the DMO of Massangam and Sightsavers. In the speeches, the various stakeholders were called upon to contribute to the realization of this initiative. In general, these speeches highlighted the raison d'etre of the Massangam Initiative, the impact of Onchocerciasis, the importance of taking the drug and a call for collaboration, good sense and appropriation.

Pr. Wanji from REFOTDE presented summarily the results of the census and screening. This assured the population that their interest is taken into account and further build trust and collaboration.

During the launching, the officials including local administrator, Sightsavers and MOH took the drug in the front stage. This is important as a boost to the community through confident building and trust. Indeed this created enthusiasm and even euphoria among the population and visibly the people showed readiness to adhere, and collaborate. It was also the lieu for us to sensitize and mobilize for the February phase of activities. A question and answer session helped clarify various issues and assured the population.

1.4. Doxycycline treatment and monitoring

1.4.1. Doxycycline treatment and eligibility

Treatment with Doxycycline is programmed for 35 days (5 weeks) with a possible extension of 1 week for catch-up in case of missed dozes within the 5 weeks. Each patient takes a capsule of Doxycycline in the morning after eating under direct observation of the CDDs (Direct Observed Therapy - DOT). Treatment involves two strategies:

- **Fix-post distribution** - where patients assemble at a one spot (food point)
- **Mobile distribution** - which involves door-to-door movement by the CDD and was adopted in case the patient did not come to the food point.

It was considered that children from 9 years and above, non-pregnant and non-breastfeeding womens and those who do not take medications for chronic health conditions

such as diabetes, epilepsy, hypertension, tuberculosis, HIV/AIDS or having other severe health conditions were eligible to take Doxycycline.

A general start date for all the communities was Tuesday the 12th, but due to the purpose of launching, some people (4 patients) and part of Mankankoun community started treatment. Those who did not start on that Tuesday were given a maximum of 7 days to start and their individual start dates noted. These people have one additional week at the end of the 5th week to make-up for lost days.

1.4.2. Monitoring and Supervision

Monitoring and supervision are carried out at different levels (Table 3), and this is to ensure that registers are filled correctly, monitor for side effects, identify challenges and measures to address them and ensure food supply is not interrupted.

At the beginning, two cases of resistance were notified but with the joint effort of REFOTDE and Sightsavers, these were handled through communication. Apart from light headache which one woman complained, no other complains were recorded on the field.

Table 3: Different levels of supervision and the persons involved

**Level of Supervision**	**Person involved**
Front line health implementer	CoCs of hotspot communities
REFORDE	Research Officers
District team	Health Area Chief, District Data Manager and DMO
Regional team	Regional Delegate and Regional Focal Person for NTDs
Central team	National Coordinator for Onchocerciasis
Sightsavers team	Research Officer

2. Treatment statistics

A total of 170 OV positive cases were registered in the 3 hotspot communities for the first phase of Doxycycline treatment, with 125 eligible and 45 non-eligible due to either pregnant, breast feeding, sick or under aged (Table 4). Of the 125 eligible, 120 were enrolled for treatment. Amongst the sicknesses recorded were epilepsy, HIV/AIDS, and Hepatitis.

Table 4: Summary statistics for 3 days (Monday – Wednesday) doxycycline treatment at Massangam

SUMMARY STATISTICS FOR LAUNCHING OF DOXY TREATMENT AT MASSANGAM, WEST REGION, CAMEROON												
Community	No of Oncho mf positives	Persons not eligible for Doxy					Total No not eligible	Total Eligible	No enrolled for treatment	Drop out	Unidentified	No not enrolled for treatment
		Pregnant	Breast Feeding	Sick	Travelled	Under Aged						
Makouopsap	53	3	3	5	1	5	17	36	34	0	0	0
Mankakoun	103	3	11	6	4	0	24	79	76	1	1	1
Njingouot	14	2	0	0	2	0	4	10	10	0	0	0
TOTAL	170	8	14	11	7	5	45	125	120	1	1	1

The above table is still to be cleaned after REFOTDE visits the field for monitoring.

3. Challenges

Some challenges were noticed during this first phase of doxycycline treatment and measures taken to minimize:

- **Internal and external movement** – Some people who were screened in one community and results positive happens to be residing in another community during doxycycline treatment, either temporarily or permanently and others completely moved out of the village. This could lead to patient lost to follow up and cases of default. In order to resolve this problem, a list of these persons and the new community where they are residing was established. This list was handed to the CDD in charge of the new community to add to his/her list of patients. Whenever the patient returns to his/her community, the CDD of that community will continue the follow-up. In the case of permanent relocation, the monitoring team together with the CDD concerned try to contact the patients and/or family. It was agreed that if these patients return to the village within 7 days of treatment, they will be enrolled for this round of doxycycline treatment and followed up. If they come back after 7 days into treatment, they will be scheduled for the next round of treatment.
- **Resistance**– This first round of doxycycline treatment recorded two cases of resistance that was overcome after further sensitization by the CDD as well as the monitoring team.
- Some patients wanted money for food to be handed to them. It was made cleared that this practice is not allowed.

4. Next step

- Continuous monitoring, mobilisation and sensitization
- Insecticide susceptibility and feasibility verification
- Ground larvaciding

5. Conclusion

The first phase of doxycycline treatment has recorded some degree of success due to greater level of community mobilization and sensitization. This could be seen through the inhabitants' enthusiasm to be screened and willingness to take doxycycline even when they are not OV positive. In addition, the local authorities have been completely involved giving the community a sense of increase participation and commitment. Furthermore, the lessons learned from COUNTDOWN has been used in Massangam, leading to better management of challenges that arise. The next round of doxycycline treatment would certainly see much participation not only because of a larger number of activities (test and treat and MDA) but also because this first phase has paved the way by building trust, which will allow missing cases to be caught.
