# Supplementary material for: Acceptability of test and treat with doxycycline against Onchocerciasis in an area of persistent transmission in Massangam Health District, Cameroon
Source: PLoS Negl Trop Dis. 2023 Apr 5;17(4):e0011185. doi: 10.1371/journal.pntd.0011185 (PMC10075443; doi:10.1371/journal.pntd.0011185)
Supplement: S3 Text — (PDF) [file pntd.0011185.s003.pdf]

Preparation meeting

Dates: 13th August, 2018.

Attendance: Prof Kamgno Joseph, Prof Boakye, Dr Rogers Nditanchou, Dr Laura Senyonjo, Mrs Kareen Atekem,

Action points – AIS planning meeting and field visit

1. **Parasitology**
   - Screen all eligible people in the focus of high transmission and these include:
     - Previously screened positives and received doxy treatment – rationale being that this will increase chances of killing the macrofilaria that might have not have been killed during the first treatment; and also to evaluate the impact of the first doxy treatment.
     - Previously screened negatives (those who did not receive IVM and those who received IVM) – rationale being that they could be negative due to IVM intake or that they had very few mfs which were not detected in the first screening
     - New inhabitants and those who were not present /refuse/ ineligible during first screening
   - Consider using Ov-16 for first-line screening (depending on availability – timeliness and in quantity) and those positive for OV will further be screened by skin snip microscopy. However, this will be done at end line, and still to decide whether RDT or ELIZA.
   - Not recommended to use only Ov-16 for screening due to sensitivity issues – many positives therefore leading to a large number of people still to have skin snip microscopy which will not be cost effective or reduce much the number who still require the more painful biopsy.
   - To improve coverage of screening:
     - Individual feedback will be given as appropriate.
     - Slide will be read after 1-2 hours and if positive, the individual concern will be invited to see the mf under the microscope together with the community leaders so as to motivate others to come for test. Negative

slides, as well as the positive slides will be read after 24hours and the result provided to them individually

    - The test will be done progressively i.e. community after community to allow persuading message from first point above to diffuse and encourage people in the community
    - Develop new messages together with social scientist on how to better communicate or sensitize this population to be screened, and to receive and adhere to treatment. CRFiLMT to put us in contact with social scientist team
    - Community meetings with local chiefs in the presence of the Administrative authorities to increase sensitization. Make the local authorities and the community feel important. Use individuals with success stories to share their experiences. Present results obtained in previous rounds during meetings. Revise CDD selection process so to as to take into account all the selection criteria
    - Conduct exhaustive census including the Bororo camps that were/have not been covered during previous census and screening. Sightsavers to lead on this. Select a Bororo person to be as a liaise and associate the person with CDD. Also census temporary fishing or farming camps.
- CRFiLMT to finalize and share parasitological protocol together including flow chart, detailed work plan and timeframe for screening and treatment, and budget, by Friday 24th August 2018.
- IVM treatment and doxy treatment – plan with program team for IVM, training and use same CDDs to distribute both IVM and Doxy. Distribute Mectizan and Doxy simultaneously
- CDD motivation – bags and T-shirts, hats
- Qualitative assessment of acceptability and feasibility of test and treat in nomadic and settled communities after next round, built into the large acceptability and tolerability study

2. Entomology / larviciding

- Conduct larvicding in the dry season – coincide with 5 -6 months when chemotherapy effects conducted in September will have started to wain, thus

providing maximum effect of vector control; cost-effective due to less number of breeding sites to larvicide. Ideal time for larviciding will be January-February 2019. This assumes high coverage of IVM and test and treat is achieved.

- Conduct sensitivity of larvae to temephos at 2-3 breeding sites – to ascertain susceptibility of larvae at different breeding sites to temephos. Ensure larvae collected from across the breeding site.
- Re-prospect at previous sites and prospect for other new sites especially around the Kim River; 3 days before starting larviciding.
- Use human landing method for fly catching
- Prospect previous breeding sites for larvae (Mbam and Nja) and for new breeding sites (Kim) 3 days before starting larviciding. Conduct prospection in the dry season prospection (December 2018) around river Noun (Bangou bridge) to identify breeding sites for larviciding and entomology data
- A total of 5 larviciding sites to be treated during this phase 2 – 2 on Nja, 2 on Mbam and 1 on Kim. Possibly another site on Noun
- Conduct river parameter measurements to determine the flow rate and hence quantity of abate needed at each time treatment is to be effectuated at each larviciding site; and do all treatments at all the sites on the same day **for sites that are closer**
- Quantity of abate – consider maintaining the previous quantities and adding some estimates to cover for the additional breeding sites. Follow up on other suppliers of temephos
- Monitor larvicidal activity by checking all the breeding sites one day after larviciding; Fly collection once every week, the day prior to treatment to understand the trend of fly population weekly following larviciding
- Hydro-biological monitoring not needed since the impact on aquatic life will be negligible giving the amount of larviciding; but will continue to follow-up with local fishermen as to any changes in fish stocks or deaths
- Entomology team to draft and share an entomology work plan and timeframe, as well as budget (rates and mode of payment). Two weeks

In all action points for entomology

**Activity**	**Where**	**When**	**Note**
Detail work plan and budget including end line		By the end of September	Due by August to enable submission for consideration in next year budget
Purchase temephos	Locally and/or abroad	Before December ending	Quantity 1000L<br>May be added as needed
Transmission assessment	Noun	December 2018	If positive, may be considered for larviciding alongside the previous sites
	All sites	End line Sept to Nov 2019	End line will be done before MDA. Consider shifting the MDA round
Flies monitoring/larvicide monitory	Larvae monitoring, 1 day after each larviciding	All the larviciding side	This will help explain any increase in fly population during larviciding
	Flies catches<br>1 day before each weekly larviciding	All larviciding sites	This monitors overall effect of larviciding

3. **End-line parasitology and Entomology**
   - Parasitology – 5-6 months after the last round of MDA (February – March 2019) which could be in Sept – Oct 2019. Consider shifting IVM to October for the 3 HAs after end line
   - Entomology – plan conducting in August – Oct 2019, 2 days/week for 3 months
4. **Other points to consider**

- Ethical clearance to be renewed for phase 2
- Change of PI and adding Co-Is list

In summary:

**Activity**	**Who**	**Timeline**	**Comments**
**DOXY TEST AND TREAT**			
Re-census (including Mbororo camps and other small settlements e.g fishermen/farming camps)	SSI/MOH	August/Sept 2018	Evaluation of acceptability of test and treat in stable and nomadic groups
Test and treat with doxy	CRFilMT	Sept 2018	Need a treatment decision flowchart of implementation
**IVM THIRD ROUND**			
Treatment with IVM	SSI/MOH	Sept 2018	Carry out in the three HAs, all those ineligible to doxy will receive MDA, SSI to provide a census list to the screening team This census will be collated from previous Census (REFOTDE, MDA census and special census of the nomads)
**ACCEPTABILITY STUDY**			
Evaluation of acceptability of doxy test and treat and IVM biannual, comparison settled and nomadic communities	SSI	Nov 2018	Need to evaluate strategy in nomadic communities, are they systematic non-compliers and why, are they infected and did our approach to reach them work. How can the strategy be improved
**LARVICIDE**			
Transmission assessment on the Noun	Entomology team	December 2018	To determine if there are breeding sites and transmission potential – in order to inform whether need to larvicide
Re-prospect previous breeding sites	Entomology team	January 2019	Confirm if previous sites still have larvae and double check no new sites missed
Susceptibility testing on River Nja and River Mbam	Entomology team	January 2019	Full susceptibility test
Conduct river parameters	Entomology team	January 2019	Week before each planned larviciding and at every larviciding point
Larviciding at 5 sites – 2 on the Mbam and 2 on the Nja, and 1 on Kim (if prospection in Noun is positive, add sites)	Entomology team	Feb-March 2019	Do we need to larvicide on the Noun? Plan to do a transmission assessment in Dec Full 10 week treatment

Evaluate larvae in river and fly biting rates	Entomology team	Feb-March 2019	1 day a week, day prior to treatment (allows fly population time to recover after larvicide).<br>Prospection larvae 1 day after larvae
**IVM FOURTH ROUND**			
Census and treatment		March 2019	
**END LINE EVALUATION**			
Entomology		August/Sept/Oct 2019	
Parasitology		Oct 2019	Need to confirm that IVM round planned for Sept can be delayed to end Oct
	**OTHERS**		
Collect and examine the remaining 3 weeks of flies captured during monitoring of last larviciding			
