# Supplementary material for: Acceptability of test and treat with doxycycline against Onchocerciasis in an area of persistent transmission in Massangam Health District, Cameroon
Source: PLoS Negl Trop Dis. 2023 Apr 5;17(4):e0011185. doi: 10.1371/journal.pntd.0011185 (PMC10075443; doi:10.1371/journal.pntd.0011185)
Supplement: S4 Text — (PDF) [file pntd.0011185.s004.pdf]

Focus Group Discussion (FGD) Guide

Assessment of Acceptability and Effectiveness of doxycycline test and treat strategies and annual ivermectin distribution in semi-nomads in Massangam Health District

- Nomad community (adults: men and women and mixed)
- Settled community (adult: men and women and mixed)

Introductory Remarks

Good morning /afternoon, my name is ....(moderator) and my colleague is…………(note taker). We are here on behalf of the Ministry of Health and we are conducting a study on health issues in this district/LGA. The information we are collecting will help the MOH to formulate actions that will enhance the health outcomes on-going prevention and control activities in this district/LGA. You have been selected to participate in this Focus Group Discussion (FGD) because we feel your views will help in the MOH planning. We therefore, kindly request you to share your honest experiences/opinions on these issues. Participation in this discussion is voluntary. You are free not to respond to any issues you feel uncomfortable with. However, we wish to assure you that the information you give us shall be kept confidential and will only be used for purposes of this study. The information will not be linked to any participant. Our discussion will last approximately one hour during which everybody will have an opportunity to contribute. So please speak one at time and feel free to express your views even if you feel that they are different from what other participants think.

Do you have any questions or comments before we proceed?

Moderator: In case of any questions, please try to address them before proceeding.

I also wish to kindly request you to allow me tape record this discussion so that I can capture everything we discuss.

Moderator: In case participants refuse to be tape-recorded, ignore the recorder and proceed with the discussion as the note-taker writes down verbatim as much as she/he can.

IDENTIFIERS

Community		
Population (indicate nomads or settled)		
Total Number of participants	Male =	Female =

1. **Can you tell me the health problems you encounter in your community?**

- What diseases/illness - if onchocerciasis is not cited talk about it onchocerciasis and ask if they know about.
- Who/where/how do you resolve these health issues?

- **What are the barriers to accessing this health care?**
- What health interventions do you usually receive here- how and by who?

2. **Now let talk about onchocerciasis – what do you know about it?**

- What is it?
- Does it affect you community? Who (men, women, children etc) does it affect and how?
- How do people get the onchocerciasis?
- How do you treat onchocerciasis in the community? If CDTI is not mention, talk about.

3. **Tell me about Mectizan distribution (CDTI) in your community (nomad)**

- How is it done? Who gets it? How often?
- Where and who are involve (district) – do you think about them?
- Who received IVM in community and why?
- Who does not received IVM and why?
- How do you feel about /view the Mectizan distribution?
- What do you like about it and why?
- What do you not like about it and why?
- How can the issues been resolved? How can the distribution been improved?

Ok let's now talk about the recent testing and treating of onchocerciasis in your community. Let start with the testing.

4. **Can you tell me about the testing, when you were invited for your skin to be examined?**

- Who in the community get tested and why?
- Who in the community did not get tested and why?
- How was the result communicated to you?
- What was your feeling of the testing and result communicated to you after testing?
- What do you like about the testing and why?
- What do you not like about the testing and why?
- What can be improved about the testing?

5. **Let now let us talk about the treatment. Tell about the treatment after you were tested?**

- Who received the treatment in the community?
- Who did not received the treatment in the community and why?
- How was the drug given?
- What were the challenges you face taking the drug and how did you resolve them?
- What is your feeling/view about the treatment?
- What was good about the treatment and why?
- What was bad about the treatment and why?
- How can the treatment be improved? What need improvement?

Before we end, lets now talk and your community so that we know who to contact for in community (nomads)

6. **Tell me about how your community is rule/organise/manage.**
- Who made decision in your community from the family to the bigger community?
- Who communicate information in the community?
- How are information from the health district for examine passed to the community?
- How do you interact with the settled community? What you do together?
- How would you describe your relationship with the settled community?
- How is conflict in the community resolve?
- What do people in your community do (women, men and children)?
- Who usually move out of the community?
- Why/how/when/where do they travel to?
- How can your community better be involve in IVM MDA?
- How can you community better be involve in testing and treating of onchocerciasis?

7. **Recommendations. As we are ending, based on your various opinions what will be the most important thing to do in order to improve:**
- Mectizan distribution
- Testing and treating of onchocerciasis with doxycycline

Conclusion

This is the end of the discussion. Do you have any question? (Answer their questions appropriately).

Thank you very much for letting us know your views. We will use it in improving health intervention in your community.

Focus Group Discussion

Assessment of Acceptability and Effectiveness of doxycycline test and treat strategies and annual ivermectin distribution in semi-nomads in Massangam Health District

- Those not involve in mf screening Nomad community

Introductory Remarks

Good morning /afternoon, my name is ....(moderator) and my colleague is............(note taker). We are here on behalf of the Ministry of Health and we are conducting a study on health issues in this district/LGA. The information we are collecting will help the MOH to formulate actions that will enhance the health outcomes on-going prevention and control activities in this district/LGA. You have been selected to participate in this Focus Group Discussion (FGD)

because we feel your views will help in the MOH planning. We therefore, kindly request you to share your honest experiences/opinions on these issues. Participation in this discussion is voluntary. You are free not to respond to any issues you feel uncomfortable with. However, we wish to assure you that the information you give us shall be kept confidential and will only be used for purposes of this study. The information will not be linked to any participant. Our discussion will last approximately one hour during which everybody will have an opportunity to contribute. So please speak one at time and feel free to express your views even if you feel that they are different from what other participants think.

Do you have any questions or comments before we proceed?

Moderator: In case of any questions, please try to address them before proceeding.

I also wish to kindly request you to allow me tape record this discussion so that I can capture everything we discuss.

Moderator: In case participants refuse to be tape-recorded, ignore the recorder and proceed with the discussion as the note-taker writes down verbatim as much as she/he can.

IDENTIFIERS

Community	
Population (indicate nomads or settled)	
Total Number of participants	Male = Female =

1. **Can you tell me the health problems you encounter in your community?**
- What diseases/illness - if onchocerciasis is not cited talk about it onchocerciasis and ask if they know about.
- Who/where/how do you resolve these health issues?
- **What are the barriers to accessing this health care?**
- What health interventions do you usually receive here- how and by who?

2. **Now let talk about onchocerciasis – what do you know about it?**
- What is it?
- Does it affect you community? Who (men, women, children etc) does it affect and how?
- How do people get the onchocerciasis?
- How do you treat onchocerciasis in the community? If CDTI is not mention, talk about.

3. **Tell me about Mectizan distribution (CDTI) in your community (nomad)**
- How is it done? Who gets it? How often?
- Where and who are involve (district) – do you think about them?
- Who received IVM in community and why?
- Who does not received IVM and why?
- How do you feel about /view the Mectizan distribution?

- What do you like about it and why?
- What do you not like about it and why?
- How can the issues been resolved? How can the distribution been improved?

Ok let's now talk about the recent testing and treating of onchocerciasis in your community. Let start with the testing.

4. As you were not tested we would like to hear your views about the testing?

- Where we you during the screening?
- What was your feeling of the testing?
- What do you not like about the testing and why?
- What can be improve about the testing?
- What are the reasons people did not get tested?

Before we end, lets now talk and your community so that we know who to contact for in community (nomads)

5. Tell me about how your community rule/organise/manage.

- Who made decision in your community from the family to the bigger community?
- Who communicate information in the community?
- How are information from the health district for examine passed to the community?
- How do you interact with the settled community? What you do together?
- How would you describe your relationship with the settled community?
- How is conflict in the community resolve?
- What do people in your community do (women, men and children)?
- Who usually move out of the community?
- Why/how/when/where do they travel to?
- How can your community better be involve in IVM MDA?
- How can you community better be involve in testing and treating of onchocerciasis?

6. Recommendations. As we are ending, based on your various opinions what will be the most important thing to do in order to improve:

- Mectizan distribution
- Testing

Conclusion

This is the end of the discussion. Do you have any question? (Answer their questions appropriately).

Thank you very much for letting us know your views. We will use it in improving health intervention in your community.

In-depth Interview (IDI) Guide

Assessment of Acceptability and Effectiveness of doxycycline test and treat strategies and annual ivermectin distribution in semi-nomads in Massangam Health District

- CDD

Introductory Remarks

Good morning /afternoon, my name is..........**(interviewee).** We are here on behalf of the Ministry of Health and we are conducting a study on health issues in this district. The information we are collecting will help the MOH to formulate actions that will enhance the health outcomes on-going prevention and control activities in this district. You have been selected to participate in this IDI because we feel your views will help in the MOH planning. I therefore, kindly request you to share your honest experiences/opinions on these issues. Participation in this discussion is voluntary. You are free not to respond to any issues you feel uncomfortable with. However, I would like to assure you that the information you give us shall be kept confidential and will only be used for purposes of this study. The discussion will last approximately thirty minutes.

Do you have any questions or comments before we proceed?

Interviewer: In case of any questions, please try to address them before proceeding.

I also wish to kindly request you to allow me tape record this discussion so that I can capture everything we discuss.

Interviewer: In case the participant refuses to be tape-recorded, ignore the recorder and proceed with the discussion and take all notes.

IDENTIFIERS

Community	
Population (nomads or settled community)	
Title/Position	
Sex	Male (1) Female (2)
Length of stay in the area	
Duration and area as CDD	

MAIN COMMUNITY AND NOMADS

1. How long have you been CDD in this community?
2. What is your role in distributing Mectizan
3. Which area and population do you cover and how would you rate the perfomance?

4. Can you tell me the health problems encounter in those community?

- What are the main diseases/illness affecting the community
- Who/where/how do the community resolve these health issues?
- What are the barriers to accessing this health care?
- What health interventions do the community usually receive here- how and by who?

Let's now talk about awareness of onchocerciasis (ask main then, nomads)

5. How much does the community in which you distribute Mectizan know about onchocerciasis?

- Does the community feel it is affected by onchocerciasis?
- Who (men, women, children etc) does it affect and how?
- How does the community think they get onchocerciasis?
- How does the community treat the onchocerciasis?
- What is your feeling about the level of awareness of the onchocerciasis in the community in which you distribute Mectizan? High or low and why?
- What can be done to improve awareness?

IVM Mass Drug Administration (nomads)

6. How is Mectizan distributed in this community?

- How often is Mectizan distributed in the nomad community?
- How is the community involve?
- What support do you get form the district?
- What support do you get from the community?

7. How much does the nomad community know about Mectizan distribution?
- How do they feel about the Mectizan distribution?
- What is their altitude towards Mectizan distribution?
- What are the cultural/religious issues in the community affecting MDA?
- Who refuse Mectizan and why?

8. What are your feelings/views about Mectizan distribution in the nomads
- Views on census - What are the challenges of census in the nomad community?
- Views on sensitization prior to distribution (good and bad/problems and why)
- Views on distribution (good and bad/problems and why)
- Who receive Mectizan in the community?
- Who does not received Mectizan in the community and why?
- What are the major challenges you face distributing Mectizan in this community?
- What do you suggest for improvement?

You were involve in the recent testing and treating with doxycycline. Let talk about it now. Let start with the testing.

9. Tell me about the recent screening in which skin were examined?
- How were you involve?
- Who in the community get tested and why?
- Who in the community did not get tested and why?
- How was the result communicated and how did they feel about it?
- How did people in the community feel about the testing? What was good or bad according to them and why?
- What do you like about the testing and why?
- What do you not like about the testing and why?
- What were the challenges face during the testing?
- What can be improve about the testing?

Let's now talk about the treatment with doxycycline after testing.

10. Tell me about the treatment after testing?
- Who received the treatment in the community?
- Who did not received the treatment in the community and why?
- Who were the people missing doses and why?
- How was the drug given?
- What were the challenges taking the drug and how did you resolve them?
- What is your feeling/view about the treatment?
- What was good about the treatment and why?
- What was bad about the treatment and why?

- How did the treatment suit around daily activity – yours and that of the community?
- How can the treatment be improve? What need improvement?

11. Recommendations. As we are ending, based on your various opinions what will be the most important thing to do in order to improve:

- Census in the nomad
- Sensitization and mobilisation in nomad
- Mectizan distribution in the nomads
- Screening for mf in the skin by skin snip
- Treating with doxycycline

Conclusion

This is the end of the interview. Do you have any question? (Answer their questions appropriately).

Thank you very much for letting us know your views. We will use it in improving health intervention in your community.

In-depth Interview (IDI) Guide

Assessment of Acceptability and Effectiveness of doxycycline test and treat strategies and annual ivermectin distribution in semi-nomads in Massangam Health District

- MoH

Introductory Remarks

Good morning /afternoon, my name is..........**(interviewee).** We are here on behalf of the Ministry of Health and we are conducting a study on health issues in this district. The information we are collecting will help the MOH to formulate actions that will enhance the health outcomes on-going prevention and control activities in this district. You have been selected to participate in this IDI because we feel your views will help in the MOH planning. I therefore, kindly request you to share your honest experiences/opinions on these issues. Participation in this discussion is voluntary. You are free not to respond to any issues you feel uncomfortable with. However, I would like to assure you that the information you give us shall be kept confidential and will only be used for purposes of this study. The discussion will last approximately thirty minutes.

Do you have any questions or comments before we proceed?

Interviewer: In case of any questions, please try to address them before proceeding.

I also wish to kindly request you to allow me tape record this discussion so that I can capture everything we discuss.

Interviewer: In case the participant refuses to be tape-recorded, ignore the recorder and proceed with the discussion and take all notes.

IDENTIFIERS

Community	

Title/Position	
Sex	Male (1) Female (2)
Length of stay in the community	
Duration in the position	

1. How long have you been in this position?
2. How long have your been involve in Mectizan distribution?
3. Can you tell me the health problems encounter in the community? (settled community then Nomads)

- What are the main diseases/illness affecting the community
- Who/where/how do the community resolve these health issues?
- What are the barriers to accessing this health care?
- What health interventions do the community usually- how and by who?

Let's now talk about awareness of onchocerciasis (ask settled then, nomads)

4. How much does the community know about onchocerciasis?

- Does the community feel it is affected by onchocerciasis?
- Who (men, women, children etc) does it affect and how?
- What is your feeling about the level of awareness of the onchocerciasis in the community? High or low and why?
- What can be done to improve awareness?

IVM Mass Drug Administration (nomads)

5. How is Mectizan distributed in the nomad community?

- How are CDD selected?
- What support do the CDD get?
- How often is Mectizan distributed in the nomad community?
- How is the community involve?
- What are the gender/cultural issues that may be involved?
- What are the strategies to reach the nomads population?
- Which community misses Mectizan and why?
- Are they people in community who misses Mectizan and why?

6. How much does the nomad community know about Mectizan distribution?

- How do the nomads feel about the Mectizan distribution?
- What is their altitude towards Mectizan distribution?

7. What are your feelings/views about Mectizan distribution in the nomads

- Views on census - What are the challenges of census in the nomad community?
- Views on sensitization prior to distribution (good, bad & issues and why)
- Views on distribution (good, bad & issues and why)
- Who receive Mectizan in the community and why?

- Who does not received Mectizan in the community and why?
- What are the major challenges in distributing Mectizan?
- What do you suggest for improvement?

You were involve in the recent testing and treating with doxycycline. Let's talk about it now. Let's start with the testing.

8. **Tell me about the recent screening in which skin were examined?**

- How were you involve?
- How were people sensitize and mobilise for the testing?
- Who in the community get tested and why?
- Who in the community did not get tested and why?
- What is your view of the testing – good and bad and why?
- What were the challenges face during the sensitization/mobilisation and testing?
- What can be improve about the testing?

Let's now talk about the treatment with doxycycline after testing.

9. **Tell me about the treatment after testing?**

- Who received the treatment in the community?
- Who did not received the treatment in the community and why?
- Who were the people missing doses and why?
- What were the challenges taking the drug and why?
- What was good about the treatment and why?
- How did the treatment suit around your activity?
- Issues about drug availability & delivery
- How can the treatment be improve? What need improvement?

10. **Recommendations. As we are ending, based on your various opinions what will be the most important thing to do in order to improve:**

- Census in the nomad
- Sensitization and mobilisation in nomad
- Mectizan distribution in the nomads
- Screening for mf in the skin
- Treating with doxycycline

Conclusion

This is the end of the interview. Do you have any question? (Answer their questions appropriately).

Thank you very much for letting us know your views. We will use it in improving health intervention in your community.

In-depth Interview (IDI) Guide

Assessment of Acceptability and Effectiveness of doxycycline test and treat strategies and annual ivermectin distribution in semi-nomads in Massangam Health District

- Nomad community leaders
- Settled community leaders

Introductory Remarks

Good morning /afternoon, my name is...........**(interviewee).** We are here on behalf of the Ministry of Health and we are conducting a study on health issues in this district. The information we are collecting will help the MOH to formulate actions that will enhance the health outcomes on-going prevention and control activities in this district. You have been selected to participate in this IDI because we feel your views will help in the MOH planning. I therefore, kindly request you to share your honest experiences/opinions on these issues. Participation in this discussion is voluntary. You are free not to respond to any issues you feel uncomfortable with. However, I would like to assure you that the information you give us shall be kept confidential and will only be used for purposes of this study. The discussion will last approximately thirty minutes.

Do you have any questions or comments before we proceed?

Interviewer: In case of any questions, please try to address them before proceeding.

I also wish to kindly request you to allow me tape record this discussion so that I can capture everything we discuss.

Interviewer: In case the participant refuses to be tape-recorded, ignore the recorder and proceed with the discussion and take all notes.

IDENTIFIERS

Community	
Population (nomads or settled community)	
Title/Position	
Sex	Male (1) Female (2)

Length of stay in the community	

1. **How long have you been in this position?**
2. **Can you tell me the health problems encounter in the community?**

- What are the main diseases/illness affecting the community
- Who/where/how do the community resolve these health issues?
- **What are the barriers to accessing this health care?**
- What health interventions do the community usually receive here- how and by who?

Let's now talk about awareness of onchocerciasis

3. **How much does the community know about onchocerciasis?**

- Who (men, women, children etc) does it affect and how?
- How does the community get onchocerciasis?
- How does the community treat the onchocerciasis?
- What is your feelings about the level of awareness of the onchocerciasis in the community - High or low and why?
- What can be done to improve awareness?

IVM Mass Drug Administration

4. **How long have you been involve in the distribution of Mectizan in this community?**
5. **How is Mectizan distributed in this community? – tell us how it happen?**

- Who come to distribute, where and when?
- How are you and the community involve in the distribution – census, sensitization/mobilisation and distribution?
- How often is Mectizan distributed in the community?
- Who receive Mectizan in the community?
- Who does not received Mectizan in the community and why?
- How are you and community feelings about the Mectizan distribution?
- What are the challenges of census, sensitisation and distribution in the community?
- What can be improved in Mectizan distribution?

This community was involved in the recent testing and treating with doxycycline. Let'stalk about it now. Let start with the testing.

6. **Tell me about the recent screening in which skin were examined?**

- How were you and the community involved?
- Who in the community get tested?
- Who in the community did not get tested and why?
- How was the result communicated and how did they feel about it?

- How did people in the community feels about the testing? What was good or bad according to them and why?
- What do you like about the testing and why?
- What do you not like about the testing and why?
- What were the challenges face during the testing?
- What can be improved about the testing?

Let's now talk about the treatment with doxycycline after testing.

7. **Tell me about the treatment after testing?**

- How were you involve?
- How was the drug given?
- Who received the treatment in the community?
- Who did not received the treatment in the community and why?
- Who were the people missing doses and why?
- What were the challenges taking the drug and how were they resolve them?
- What is your feeling/view about the treatment?
- What was good about the treatment and why?
- What was bad about the treatment and why?
- How did the treatment suit around daily activity of the community?
- How can the treatment be improved? What need improvement?

8. **Recommendations. As we are ending, based on your opinion what will be the most important thing to do in order to improve:**

- Census
- Sensitization and mobilisation
- Mectizan distribution
- Screening for mf in the skin by skin snip
- Treating with doxycycline

Conclusion

This is the end of the interview. Do you have any question? (Answer their questions appropriately).

Thank you very much for letting us know your views. We will use it in improving health intervention in your community.
