# Supplementary material for: Acceptability of test and treat with doxycycline against Onchocerciasis in an area of persistent transmission in Massangam Health District, Cameroon
Source: PLoS Negl Trop Dis. 2023 Apr 5;17(4):e0011185. doi: 10.1371/journal.pntd.0011185 (PMC10075443; doi:10.1371/journal.pntd.0011185)
Supplement: S1 Table — (PDF) [file pntd.0011185.s005.pdf]

S 2. Table showing timeline of implementation of Alternative Treatment Strategies package (ground larviciding, biannual ivermectin mass drug administration and test and treat with doxycycline.

	**2017**	**2017**	**2017**	**2017**	**2017**	**2017**	**2018**	**2018**	**2018**	**2018**	**2018**	**2018**	**2019**	**2019**	**2019**
Suscept. test	June														
Ivm MDA 1		Aug*													
Test 1			Sept												
Larviciding 1				Oct	Nov	Dec									
Treatment 1						Dec	Jan								
Ivm MDA 2								March							
TCS									May						
Mop-up										July					
Ivm MDA 3											Sept				
Test 2											Sept				
Treatment 2											Sept				
Qual study												Nov			
Larviciding 2													Feb	March	April
Ivm MDA 4														March	

Ivm MDA: Ivermectin Mass Drug Administration. Four rounds were implemented – 1, 2, 3, 4; test is skin snip test conducted twice – 1 and 2; treatments were conducted twice following test. Ground larviciding of productive breeding sites was conducted twice – 1 and 2. * Test 2 weeks after Ivm MDA 1.
