# Supplementary material for: Acceptability of test and treat with doxycycline against Onchocerciasis in an area of persistent transmission in Massangam Health District, Cameroon
Source: PLoS Negl Trop Dis. 2023 Apr 5;17(4):e0011185. doi: 10.1371/journal.pntd.0011185 (PMC10075443; doi:10.1371/journal.pntd.0011185)
Supplement: S2 Table — (PDF) [file pntd.0011185.s006.pdf]

Supplement. 3. Doxycycline treatment register

STRATEGIE ALTERNATIVE POUR L'ELIMINATION DE L'ONCHOCERCOSE

FICHE DE TRAITEMENT A LA DOXYCYCLINE

Nom et prénom: ___________________________________

District de Santé:__________________________

Code Individuel

☐☐ ☐☐☐☐ ☐☐

Gendre: M ☐ F ☐ Age:____ ans

Aire de Santé:____________________________

Adresse/contact:____

Communauté:_________________________

Date de début de traitement ______/________/________

Région:____________________________

Nom de relais communautaire RC /DC______________________________

Souffrez-vous de maladie(Diabètes, hypertension, épilepsies, tuberculoses, VIH-SIDA, maladie cardio-vasculaire , asthme ?: Oui ☐ Non ☐, Si oui référez le patient au centre de santé le plus proche

Pour les femmes : Etes-vous enceinte ? Oui ☐ Non ☐ Allaitez-vous Oui? ☐ Non ☐

Jours / Semaines	1	2	3	4	5	6	7	Remarques / commentaires
**Semaine 1**								
**Semaine 2**								
**Semaine 3**								
**Semaine 4**								
**Semaine 5**								
**Semaine de rattrapage**								

Précaution / étapes à suivre avant la prise de Doxycycline

- Ne pas donner à une femme enceinte/allaitante ou un enfant moins de 9 ans
- Manger avant la prise de Doxycycline
- Si vous êtes en train de prendre un autre traitement, informer le DC
- Avalez **une (1)** capsule par jour si vous-êtes éligible

** Cocher la case correspondante ci-dessus après la prise de médicament

Effets secondaires : ____ *En cas de effet secondaire persistant contactez l'infirmier en charge*

Jours / Semaines	1	2	3	4	5	6	7
**Semaine 1**							
**Semaine 2**							
**Semaine 3**							
**Semaine 4**							
**Semaine 5**							
**Semaine de rattrapage**							

NB: Ecrivez tout effet secondaire dans la case correspondante ci-dessus.

1. Diarrhée 2. Nausée 3. Vomissement 4. Maux de tête 5. Démangeaison/éruption cutanée 6. Autres (précisez) ______________________
