# Supplementary material for: Acceptability of test and treat with doxycycline against Onchocerciasis in an area of persistent transmission in Massangam Health District, Cameroon
Source: PLoS Negl Trop Dis. 2023 Apr 5;17(4):e0011185. doi: 10.1371/journal.pntd.0011185 (PMC10075443; doi:10.1371/journal.pntd.0011185)
Supplement: Attachment — Submitted filename: PNTD-D-22-01137.pdf [file pntd.0011185.s008.pdf]

PLOS Neglected Tropical Diseases

Acceptability and effectiveness of test and treat with doxycycline against Onchocerciasis in an area of persistent transmission in Massangam Health District, Cameroon

--Manuscript Draft--

**Manuscript Number:**	PNTD-D-22-01137
**Full Title:**	Acceptability and effectiveness of test and treat with doxycycline against Onchocerciasis in an area of persistent transmission in Massangam Health District, Cameroon
**Short Title:**	Acceptability of test and treat with doxycycline against Onchocerciasis
**Article Type:**	Research Article
**Keywords:**	Test and treat; doxycycline; onchocerciasis; acceptability; effectiveness
**Abstract:**	Alternative treatment strategies against onchocerciasis were implemented in Massangam Health District of Cameroon in 2017 and 2018. Implementation included two rounds of test and treat with doxycycline (TTd) leading to a significant prevalence reduction from 35.7% to 12.3% (p<0.001). TTd involved microscopic examination for microfilaria (mf) in skin biopsy and those infected were offered a 35-day course of doxycycline 100 mg, delivered by community-directed distributors (CDDs). Effectiveness and acceptability of TTd were evaluated based on TTd data, field experience and qualitative assessment. Findings revealed 54% of eligible population participated in each round; but the second round had a higher number of participants as newcomers and previous non-participating residents participated as trust increased. Semi-nomadic population participated less than the settled population (OR 0.6, P=0.003) due to their remote and disperse settlements, perceived mistrust, discrimination, linguistic and cultural barriers compounded by their non-inclusion as CDDs. Previous experience of test results not being returned, mismatch of test results and symptoms, along with length of community stays, gender, and age affected acceptability of TTd. In round one, testing occurred less than three months following ivermectin treatment which likely resulted in false negatives. Less participants started treatment in round one (71% vs 83%) due to people moving away as treatment delayed. However, completion levels were high in both rounds (93% and 98%). Comparing ivermectin and doxycycline treatments, some participants stated during qualitative assessment that ivermectin is better, while others favoured doxycycline. CDDs reported that doxycycline treatment involved more effort and time. Overall, the 54% participation level is significant given the impact on the prevalence. For future improvements, it is important to explore frequently excluded populations and adapt plans to reach them; improve sensitisation; combine doxycycline and ivermectin treatments; and augment CDD compensation. Additionally, a less invasive, but sensitive test for onchocerciasis will be more acceptable.
**Additional Information:**	
**Question**	**Response**
**Financial Disclosure**<br><br>Enter a financial disclosure statement that describes the sources of funding for the work included in this submission. Review the submission guidelines for detailed requirements. View published research articles from *PLOS NTDs* for specific examples.<br><br>This statement is required for submission	This work is funded by Sightsavers. The funder had no role in study design, data collection and analysis, decision to publish, or preparation of the manuscript.

Powered by Editorial Manager® and ProduXion Manager® from Aries Systems Corporation

and **will appear in the published article** if the submission is accepted. Please make sure it is accurate.<br><br>**Unfunded studies**<br><br>Enter: *The author(s) received no specific funding for this work.*<br><br>**Funded studies**<br><br>Enter a statement with the following details:<br>• Initials of the authors who received each award<br>• Grant numbers awarded to each author<br>• The full name of each funder<br>• URL of each funder website<br>• Did the sponsors or funders play any role in the study design, data collection and analysis, decision to publish, or preparation of the manuscript?<br>• **NO** - Include this sentence at the end of your statement: *The funders had no role in study design, data collection and analysis, decision to publish, or preparation of the manuscript.*<br>• **YES** - Specify the role(s) played.<br><br>* typeset	
**Competing Interests**<br><br>Use the instructions below to enter a competing interest statement for this submission. On behalf of all authors, disclose any competing interests that could be perceived to bias this work—acknowledging all financial support and any other relevant financial or non-financial competing interests.<br><br>This statement **will appear in the published article** if the submission is accepted. Please make sure it is	The authors have declared that no competing interests exist.

accurate. View published research articles from *PLOS NTDs* for specific examples.<br><br>**NO authors have competing interests**<br><br>Enter: *The authors have declared that no competing interests exist.*<br><br>**Authors with competing interests**<br><br>Enter competing interest details beginning with this statement:<br><br>*I have read the journal's policy and the authors of this manuscript have the following competing interests: [insert competing interests here]*<br><br>* typeset<br><br>This statement is required for submission and **will appear in the published article** if the submission is accepted. Please make sure it is accurate and that any funding sources listed in your Funding Information later in the submission form are also declared in your Financial Disclosure statement.	
**Data Availability**<br><br>Authors are required to make all data underlying the findings described fully available, without restriction, and from the time of publication. PLOS allows rare	Yes - all data are fully available without restriction

exceptions to address legal and ethical concerns. See the PLOS Data Policy and FAQ for detailed information.<br><br>A Data Availability Statement describing where the data can be found is required at submission. Your answers to this question constitute the Data Availability Statement and **will be published in the article**, if accepted.<br><br>**Important:** Stating 'data available on request from the author' is not sufficient. If your data are only available upon request, select 'No' for the first question and explain your exceptional situation in the text box.<br><br>Do the authors confirm that all data underlying the findings described in their manuscript are fully available without restriction?	
**Describe where the data may be found in full sentences. If you are copying our sample text, replace any instances of XXX with the appropriate details.**<br><br>• If the data are **held or will be held in a public repository**, include URLs, accession numbers or DOIs. If this information will only be available after acceptance, indicate this by ticking the box below. For example: *All XXX files are available from the XXX database (accession number(s) XXX, XXX.*).<br>• If the data are all contained **within the manuscript and/or Supporting Information files**, enter the following: *All relevant data are within the manuscript and its Supporting Information files.*<br>• If neither of these applies but you are	The data underlying the results presented in the study are available from Rogers NDITANCHOU, rnditanchou@sightsavers.org

Powered by Editorial Manager® and ProduXion Manager® from Aries Systems Corporation

able to provide **details of access elsewhere**, with or without limitations, please do so. For example:<br><br>*Data cannot be shared publicly because of [XXX]. Data are available from the XXX Institutional Data Access / Ethics Committee (contact via XXX) for researchers who meet the criteria for access to confidential data.*<br><br>*The data underlying the results presented in the study are available from (include the name of the third party and contact information or URL).*<br>• This text is appropriate if the data are owned by a third party and authors do not have permission to share the data.<br><br>* typeset	
Additional data availability information:	

Manuscript

Click here to access/download;Manuscript;ATS_TTd_qual_final_03092022_

Title

Acceptability and effectiveness of test and treat with doxycycline against Onchocerciasis in an area of persistent transmission in Massangam Health District, Cameroon

Short title

Acceptability of test and treat with doxycycline against Onchocerciasis

Authors and affiliation

Rogers Nditanchou[1*], Ruth Dixon[2], Kareen Atekem[1], Serge Akongo[1], Benjamin Biholong[3], Franklin Ayisi[3], Philippe Nwane[4], Aude Wilhelm[2], Sapana Basnet[2], Richard Selby[2], Joseph Oye[1], Joseph Kamgno[4,5], Daniel Boakye[6], Elena Schmidt[2], Laura Senyonjo[2]

[1]Sightsavers, Cameroon Country Office

[2]Sightsavers, Haywards Heath, United Kingdom

[3]National Programme for the Fight against Onchocerciasis and Lymphatic Filariasis, Ministry of Public Health, Cameroon

[4]Filariasis and other Tropical Neglected Diseases Research Center, Yaoundé, Cameroon Yaoundé, Cameroon

[5]Faculty of Medicine and Biomedical Sciences, University of Yaoundé I, Yaoundé, Cameroon

[6]Parasitology Department, Noguchi Memorial Institute for Medical Research, University of Ghana, Legon, Accra Legon, Accra

*Corresponding author

rnditanchou@sightsavers.org

Abstract

Alternative treatment strategies against onchocerciasis were implemented in Massangam Health District of Cameroon in 2017 and 2018. Implementation included two rounds of test and treat with doxycycline (TTd) leading to a significant prevalence reduction from 35.7% to 12.3% ($p<0.001$). TTd involved microscopic examination for microfilaria (mf) in skin biopsy and those infected were offered a 35-day course of doxycycline 100 mg, delivered by community-directed distributors (CDDs). Effectiveness and acceptability of TTd were evaluated based on TTd data, field experience and qualitative assessment. Findings revealed 54% of eligible population participated in each round; but the second round had a higher number of participants as newcomers and previous non-participating residents participated as trust increased. Semi-nomadic population participated less than the settled population (OR 0.6, P=0.003) due to their remote and disperse settlements, perceived mistrust, discrimination, linguistic and cultural barriers compounded by their non-inclusion as CDDs. Previous experience of test results not being returned, mismatch of test results and symptoms, along with length of community stays, gender, and age affected acceptability of TTd. In round one, testing occurred less than three months following ivermectin treatment which likely resulted in false negatives. Less participants started treatment in round one (71% vs 83%) due to people moving away as treatment delayed. However, completion levels were high in both rounds (93% and 98%). Comparing ivermectin and doxycycline treatments, some participants stated during qualitative assessment that ivermectin is better, while others favoured doxycycline. CDDs reported that doxycycline treatment involved more effort and time. Overall, the 54% participation level is significant given the impact on the prevalence. For future improvements, it is important to explore frequently excluded populations and adapt plans to reach them; improve sensitisation; combine doxycycline and ivermectin treatments; and augment CDD compensation. Additionally, a less invasive, but sensitive test for onchocerciasis will be more acceptable.

Author summary

Doxycycline was offered to treat river blindness in Massangam health district of Cameroon after test indicating they are infected. The test, conducted twice, involved taking a small piece of skin for examination. Those infected were given doxycycline for 35 days by community volunteers. Testing and treatment were evaluated based on records, field observation and

asking the people about their feelings. Among those who were eligible for the test, 54% were tested each time. Participation was influenc[illegible] trust level and believes; and varied by sex, age, and duration of community stay. Semi-nomadic population participated less than sedentary because of their remote locations, language and cultural barriers, perceived discrimination, and mistrust of the sedentary population. Fewer people than infected started treatment first time due to delays in starting treatment. However, almost all those starting treatment completed it each time. Some beneficiaries prefer the usual ivermectin treatment. For the volunteers delivering treatment, treatment was demanding in terms of effort and time. Overall, the level of participation was satisfactory but could be improved through more sensitisation, reviewing volunteer compensation, inclusion of the frequently excluded population, developing a sensitive less invasive test, and combining doxycycline and ivermectin treatments.

Introduction

Onchocerciasis is a parasitic disease that causes skin lesions, severe itching, visual impairment and irreversible blindness (1). The main approach for control and elimination of onchocerciasis is interruption of transmission through annual Community-Directed Treatment with microfilaricide, ivermectin (CDTi). Although significant progress has been made in its control, there are still considerable challenges on the road to elimination due to very high initial or persistent transmission, starting treatment late or co-endemicity with *Loa loa* (a contraindication for ivermectin treatment) among others (2). In these situations, complementary or alternative treatment strategies (ATS) may be required to meet elimination targets. ATS are all strategies other than the usual annual CDTi and include optimised CDTi, biannual or pluriannual CDTi, complementary vector control, test and treat (T&T) and the use of alternative drugs to ivermectin, including doxycycline (3, 4).

Massangam Health District (HD) in the West Region of Cameroon is one such place where ATS is required. Findings in 2016 showed infection level by skin snip microscopy as high as 37.1% suggesting the area is not on track to elimination (5). Responding to the infection level, we implemented ATS that included biannual CDTi, ground larviciding (6, 7) and test and treat with doxycycline (TTd). An impact evaluation of these interventions showed a significant and rapid reductions in mf prevalence (from 35.7% to 12.3%; p<0.001) and Community Microfilaria Load (CMFL), from 0.66 to 0.22 mf/ss in the focal communities over a two-year period (8).

To date, TTd delivery has primarily been within research settings. This implementation in the focus of Massangam was delivered in a programmatic setting. Exploration of its acceptability, effectiveness and lessons learnt during operationalisation are essential for replication in foci encountering difficulties reaching elimination. We report findings from field operations, meeting notes, partner activity reports and qualitative assessment of the TTd. In addition, we reflect on barriers and enablers for implementation and report the solutions and adaptations developed before concluding with recommendations for future implementation.

Methods

Implementation of test and treat

TTd was conducted over two rounds, the first in July 2017 (intervention 1) and the second round in September 2018 (intervention 2). Delivery was supported by an enhanced package of programme monitoring and evaluation including detailed field notes and a mid-point qualitative evaluation. In addition, partner activity reports and meeting minutes are reviewed to summarise challenges and adjustments made during the implementation.

Following mobilization and sensitization, a census was conducted. During the census, sensitization was reinforced and every eligible individual (above the age of 8, neither pregnant nor breast feeding and without severe illness) in high infection communities was invited to a fixed location for onchocerciasis screening (test) through skin snip biopsy (8). Infected participants (presence of mf in the skin) were offered a 35-day course of doxycycline 100mg under directly observed therapy strategies by CDDs following provision of a light morning meal. Treatments were monitored for completion and side effects (detailing nature, severity, and frequency) daily by CDDs and noted in treatment registers.

Screening and treatment data were recorded on paper-based registers and then entered into Excel and imported into Stata statistical analysis software where it was cleaned and analysed. Screening participation, positivity, treatment start and course completion proportions, in each intervention were compared using logistic regression modelling between subgroups: sex, age group, community, length of stay in the community and tribe. During both implementations, researchers made direct field observations, meeting screening and treatment teams, reviewing their field notes, conducting debriefings, and adjusting implementation strategies accordingly.

Individuals were tracked between interventions by name, sex, age and community. A cumulative screening participation was calculated as the number of individuals participating in one or more intervention over the registered population recorded during both rounds.

Qualitative Assessment of Test and Treat with Doxycycline

Qualitative assessment was conducted to explore participants' experiences of TTd. Seventeen Focus Group Discussions (FGD) of six to ten people and 30 Key Informant Interviews (KII) (composition detailed in table 1) were conducted using interview guides. Participants were purposefully selected to include all identified sub population groups (youths, women, men, semi-nomadic and settled populations) from far and near locations of the communities. Key informants included community leaders as well as the implementers from different levels of the programme (Regional Neglected Tropical Diseases (NTD) focal person, District Medical Officer, Chief of Massangam Health Area, Chiefs of health facilities and community leaders). Interviews were recorded and transcribed verbatim. Transcripts were imported into Nvivo qualitative data analysis software (Version 12, QSR International Pty Ltd; www.qsrinternational.com/nvivo). Using Nvivo, they were coded by trained researcher and reviewed independently by two qualitative assessment experts, and then organised into themes and subthemes. Findings are presented alongside data from quantitative surveys, field notes and meeting reports. Appropriately de-identified data (quotes) were extracted to support the themes.

Ethical clearance for this study was obtained from the *Comité National d'Ethique de la Recherche pour la Santé Humaine* (CNERSH) in Yaoundé, Cameroon (clearance N° 2017/06/918/CE/CNERSH/SP). Written informed consent was acquired ahead of participation in any aspect of the project, as was parental consent (written) for any individual aged 17 or under.

Results

Test participation

Participation levels during intervention rounds 1 and 2 are shown in figure 1 and detailed in table 2.

In intervention 2, more residents were registered (2,380) and eligible (1,615) than in intervention 1,1921 registered and 1,211 eligible. In both intervention rounds, 54% representing 643 in round 1 and 879 participants in round 2 participated in the test. Combining both intervention rounds together in a matched dataset, in total, 3,549 individuals were registered by census. From the 3,549 total: 1,169 appeared only in intervention 1 and were not re-registered during intervention 2; 1,628 appeared only in intervention 2; 752 individuals appeared in both. Thereby, there was a turnover (increase in population) between interventions of 54.3% (1928/3549). A total of 2,503 (70.5%) were eligible age-wise for at least one of the 2 interventions and 1,433 of them were tested at least once. This equates to a cumulative testing participation level of 57.3% (1,433) of the age-eligible population. However, considering self-reported participation in round 2, 83% (1,337) participated in at least one round. Seven percent (167) of all registered during intervention 2 indicated recent arrival in the area (resident for less than 2 years)

In qualitative assessment, participants mentioned they were motivated to participate in the test for several reasons including the desire to: know their infection status; have better health; and protect others. Some respondents detailed that participation showed a sense of responsibility, solidarity, and care for community wellbeing.

> *"That (test) permitted my illness to be revealed. Therefore, for me it was good – especially that I did not have any problem following that. And everyone was happy with the result given that everyone knows that if they are positive, they will be offered treatment" (Settled community- based participant).*

Forty six percent (46%) of people did not participate. The levels of non-participation were not uniform with regards to age and community categories. In a multiple logistic regression (table 3) the likelihood of participating in the test were lower in communities of Njinjia/Njingouet (OR 0.3; p=0.001) in intervention 1, and in communities of Mankakoun (OR 0.9; p=0.001) during intervention 2 when compared to Makouopsap community (table 2). The likelihood of

participation, compared to 9-15 years age group, were significantly higher among those in the 26-40 (OR 1.5; p=0.024) and >40 (OR 2.3; p<0.001) age groups in intervention 2.

Qualitative assessment elaborated reasons for non-participation. Some youth of the settled communities felt they were not sick nor at risk and saw no reason to bear the wound, pain, or any unforeseen negative impact of the test. Reportedly, their trust and confidentiality were being influenced by speculation that testing apparatus will "shout out" all the sickness in their body audibly in public. Participants mentioned that collected skin snips are intended for malicious use to obtain mystical power. Report of previous tests with no results provided was said to be demotivating. The lack of results from tests was also cited as supporting evidence for the belief that skin samples were used to obtain mystical power. Furthermore, some participants doubted the test results; questioning that they experience symptoms of onchocerciasis as detailed in sensitisation yet have negative results.

> *"I refused…. Why are they cutting again this time around? I did not take treatment [last time]. They told me I have no illness in me. Meanwhile I know that I have illness such as eye pain, skin rashes and filaria [onchocerciasis]. For someone like me – look at my eyes, I also have skin rashes but to my greatest surprise they said I was negative. It surprised me" [semi-nomadic chief].*

To address concerns raised, adjustments were made for intervention 2 including reinforced social mobilization and sensitization using a mobile musical van to make announcements about benefits of the intervention and invite people to participate. In addition, microscopic examination of samples was done at the sampling point with immediate return of result and inviting positive participants to view live microfilariae through microscope. There was additional participation in intervention 2 evident by quantitative findings as 15% (83) of those declining to participate in intervention 1 participated in intervention 2. In addition, 64% (566) of those participating in the test were people who had not been registered in intervention 1 round despite indicating they had been resident in the study area at the time (resident for more than 2 years). One hundred and sixty-nine (169) people participated in both tests. Qualitative findings equally reflected the increased in participation.

> *"This year that they came again for the test, I was tested this time around; two days after, the result came out… they told us…" (male semi-nomad).*

> *"When people were seeing in the microscope, they were shocked to know that microbe lives in them. These made adhesion [to testing by the population] total! People like*

concrete proof. Because …. you say take this medicine and they reply, "prove to me I am sick". Them seeing the microbe under the microscope – no question anymore" (District Medical Officer).

During intervention 1, a minority semi-nomadic subgroup with a considerable population size was noticed in the study area, who appeared not to have been equitably reached for census or TTd. In intervention 2, efforts were made to reach semi-nomads, but MLR indicated semi-nomads were less likely to participate when compared to the settled (Bamoun tribe) population (OR 0.4; p<0.001, table 3). Qualitative assessment revealed semi-nomadic specific barriers to participation: their remote, dispersed and small settlements made engagement difficult; their high mobility (seasonal and daily) lead to absence during drug distribution; the lack of inclusion of semi-nomads as Community-Directed Distributors (CDDs) in routine MDA reinforced low acceptance through dis-trust; linguistic and cultural differences between CDDs and semi-nomads also contributed; limited knowledge about semi-nomads leading to their inadequate consideration during MDA programme planning; low awareness of onchocerciasis and MDA and low education (literacy) levels among semi-nomadic community members were also mentioned as barriers.

"The difficulty is more communicational. When we arrive at the health centre, we are unable to make ourselves understood by the nurses and we are also unable to understand what they are saying to us. This affects us a lot as very often the disease that we consult for is not what is being treated due to faulty comprehension between us and the nurses" [Female semi-nomad].

"I face some difficulties, I gave drug to one person [a semi-nomad], saying take one for [every] one day, instead he was taking two per day as he wanted the sick[ness] to finish quickly. He did so for 5 days. I was afraid. The difficulty now is to go and reach their place. There is problem: water, bush, all those things. You know, there was a time all road to their place were muddy and flooded. At times, the motorbike will be stuck, and you will need to trek…." [male settled community-based CDD].

Treatment participation

Twenty four percent (161) and 29% (251) of participants were found infected in interventions 1 and 2 respectively (table 2). Applying MLR, risk of infection was higher in Makakoun compared to Makouopsap (OR 2.5; p<0.001) in intervention 1. However, the risk of infection

Makakoun was lower when compared to Makouopsap during intervention 2 (OR 0.6; p=0.014). For both rounds, infection risk was higher in older age groups than the 9-15 years. Males were more often infected than female participants (OR 1.7; p =0.004 for round 1; and OR 1.6; p=0.020 for round 2) (table 3). From qualitative assessment, participants expressed satisfaction with the test clarifying their infection status and optimism they will be treated.

Treatment participation levels in the two intervention rounds are found in table 2. During intervention 1, a gap between testing and treating people resulted in a loss to follow-up as only 71% of infected participants started treatment. The census, testing and treating were streamlined during the second round, effectively reducing attrition. In intervention 2, some participants that were found positive self-reported they had positive result and received treatment during intervention 1. In response, a quality assurance of the doxycycline was performed, which was passed, ahead of the second round.

From qualitative findings, participants were appreciative of the meal support, reporting that the treatment arrangement was suitable and aided their daily activities.

> *"Every morning, we took café or "pap" [a type of corn-based food] then we took the medication, and everyone was good for the rest of the day. We were not really disturbed by the time of the treatment as it never prevented one from going about daily activities especially as the treatment points were not far from our houses. None of us were really making big efforts to go to the treatment point". [settled community-based participants].*

Furthermore, treatment participation was frequently described as a positive experience with perceived improvement to health.

> *"I took [doxycycline]. I even felt certain internal bumps in the body; but now I see external, I feel it's going out, pushing and going out…. There is no problem. But we feel that it* [doxycycline] *is pushing this disease in the body out. And I know when it's out, any moment it's going to end. When it is hiding, it will disturb years and years in the body." [Settled community-based male]*

Relating ivermectin and doxycycline treatments

Some participants compared ivermectin and doxycycline treatments with some reporting ivermectin as better than doxycycline for alleviating symptoms; meanwhile others mentioned doxycycline is better.

> *"It [ivermectin] has helped people a lot. At first, I was seeing small bumps on my body, as I consume ivermectin, I realised that the bumps have completely disappeared. Ivermectin has helped many people. So, if you want to help us, bring more ivermectin because I don't really understand the point of doxycycline" [settled community beneficiary].*

> *"…they noted changes, for example, we have someone here who constantly complaint of body itches, when he took doxycycline, it disappeared. This person was taking Ivermectin previously" [CDD].*

Programme staff expressed concern about timing between ivermectin and TTd, citing a lack of clarity on how ivermectin will impact TTd. This resulted to testing in intervention 1 being less than three months since CDTi, suspected to have contributed to low detected infection level (24%, n=643, table 2) (baseline was higher (36%, n=557) (8). This prompted catch-up round (intervention 2) where everyone was offered testing and results revealed a higher infection level than in round 1 (round 2 being 29%, n=879, table 2).

Treatment burden on the community-directed drug distributors

CDDs reported the treatment was a burden as the treatment process took their whole day over a 35-day duration, restricting their daily activities, with insufficient financial compensation.

> *"The difficulty of the CDD is that the 35 days is not easy. You are obliged to always remain on the spot….. Because the small thing [money] that they give us, does not meant that it can compensate our day of work. We can say the 35 days is sacrifice as the CDD is incapable of carrying out their own activities– go do other things, travel etc. You are obliged to be around during the 35 days and after the 35 days if there is catch-up." [Female settled community based CDD].*

During treatment, settled community based CDD struggled to reach semi-nomadic camps daily. In response, doxycycline was delivered weekly to semi-nomadic communities. Drug packets were requested for verification during each visit to ensure adherence. Although the

CDD appreciated these adaptations, they raised cases where patients misunderstood dosing, which was not promptly resolved due to the weekly visits.

> *"I face some difficulties, I gave drug to one person [a semi-nomad], saying take one for one day, instead he was taking two per day as he wanted the sick[ness] to finish quickly. He did so for 5 days. I was afraid. The difficulty now is to go and reach their place. There is problem: water, bush, all those things. You know, there was a time all road to their place were muddy and flooded. At times, the motorbike will be stuck, and you will need to trek…. The seven days was a good idea, but you are not sure if s/he has taken for the seven days…. When you give for a week and you go, upon returning, you still have two tablets left when the week has finished" [male CDD].*

Side effects of doxycycline treatment

Records of side-effects in the registers indicate that15% (13) of participants reported side-effects during intervention 1. Reports included headache, dizziness, stomach-ache, but none of them stopped treatment or sought medical attention in the hospital. During intervention 2, 25% (55) of participants reported side effects which did not necessitate stopping treatment or consultation. Similar side effects were mentioned in the qualitative assessment and there was equally no mention of consulting in health facilities.

> *"There were no side effects. Nobody came for that; nobody. I think as they ate before swallowing the drug, that is the reason; no side effect after 35 days of treatment with doxycycline. That is strange!" [District Medical Officer].*

Beneficiary feedback from the field for improvement

Improvement was proposed around gender inclusion, by selecting more female CDDs. In addition, female associations should be prioritized for sensitisation during intervention. Participants reported absenteeism as reason for some participation gaps, suggesting more time be allowed to offer more people opportunity to participate. This extended timeframe would benefit semi-nomadic communities, who are often absent because of their daily and seasonal mobility. Benefits will also apply to the settled community members who miss testing due to absence.

> *"Like those who came to claim the ivermectin from me [afterward], I could no longer [give]; I could still give with what permission? I could not. Because when they [semi-nomads] arrive, they are informed by the old ones; [and] they come to us to ask [theirs].*

Well, then there is nothing we can do ... We do not have permission [to give because distribution period is over]" [Female settled community based CDD].

Some CDDs and semi-nomads reflected that the programmatic set up or delivery model were sub-optimal for semi-nomadic populations. Semi-nomads requested that the sensitisation contents, support, channels and language are adapted to include their needs, ideally, they should be directly included in planning and implementation.

"For us Bororo [Fulani semi nomads], if you assign us this task, to one or more Bororos, it will be very easy to mobilize a lot more people. They will be able to say to theirs, in a convincing manner, what you want to pass on as message. Even those absent will be well informed, given that we know each other in detail. There are some of us that you cannot see but we know where and when to meet them and above all, how to speak to them to participate. Some of us were present at the Centre during the test but did not do [participate] because there was nobody to better explain to them the reasons for the test, the procedure, and the risk" [Female semi-nomadic participants].

Participants believed that more systematic local engagement through local chief, camp leaders and inclusion of semi-nomads as CDDs will improve participation. Outreach to semi-nomadic camps testing, moving from camp to camp, staying on site until absent residents return home if necessary, was suggested by semi-nomadic chiefs as a way of improving inclusion.

Discussion

Participation level reached 54% in each round increasing to a minimum of 57% and a maximum of 83% when both rounds are considered. The participation level was close to 60.5% obtain in Mbanga and Melong Health Districts of Cameroon where doxycycline treatments were offered without prior testing (9). An approach we did not attempt due to required cost, long duration of treatment and logistics demands (10) which impact cost effectiveness for programme. The turnover of 54% highlights the challenges of understanding the true census/denominator of the target population, especially as a significant semi-nomadic population was present in the area, whom were not systematically engaged during intervention 1. However, we believe this turnover is inaccurately excessive due to a high level of mismatch between registers of intervention 1 and 2, which was conducted manually based on location, names, age, and sex. Such mismatching led to an underestimate in cumulative population test participation over 2 rounds by inflating the denominator population. Therefore, the 57.3% is considered a

conservative minimum. However, the 46.7% remaining is still significant especially if prevalent of infection in them is higher than in those who participated - something possible as those not participating may have not been participating in usual MDA given that the same platform is used.

Previous onchocerciasis research activities that took skin snips from people in the area (11, 12) may have biased the population's self-reported past participation. Recall bias was minimised by asking participants to specify the year that tests were conducted; only responses citing the relevant year were included in analysis. Thus, participation over the two rounds is near 83%. This high participation was due to the repetition of the test, often people who refused in round 1 gained trust from talking with peers who had tested, and then accepted to participate during round 2 that also benefited from reinforced sensitization. There were also newcomers arriving into the area after round 1 and participating in round 2. Qualitative data revealed concerns surrounding health and disease status was a common motivator for test participation. In connection, TTd resistance from younger people stemmed from them feeling healthy and not needing treatment or an invasive and painful test. Quantitative findings backed up the qualitative findings, revealing older age group testing participation was more likely than the younger population (OR >1 and $p<0.05$). This study found high infection (OR >1 and $p<0.05$) among older age categories, who are therefore more likely to experience onchocerciasis-related signs and symptoms driving increased test acceptance (13).

Low participation amongst the semi-nomadic population became apparent during the implementation. Despite adjustments to include semi-nomads, the relative participation level was 40% lower compared to the settled population, this highlights the severity of equity issues. Qualitative findings confirmed and explored reasons for inequity, revealing mistrust, ill-adapted sensitisation, programme reach issues linked to use of CDDs from settled community, their frequent mobility and their remotely dispersed small settlements. Inequity factors are compounded by inherent programmatic issues, not adequately incorporating semi-nomadic challenges into programme strategies. The semi-nomads' mistrust of the settled population is rooted in conflict and discrimination frequently stemming from land access conflict. Not reaching these semi-nomads threatens the impact of the intervention and its sustainability. Systematic programme planning including semi-nomads, reinforced engagement, adaptations of sensitisation (channels, language, and content), inclusion of semi-nomadic CDDs as well as outreach to their settlements are strategies to consider.

Concerns surrounding confidentiality and trust of the test were reported among the wider population. Qualitative assessment reveal beneficiaries' perspectives such as getting a negative result when symptomatic, impacted their acceptability of the programme. This mismatch has been partially due to testing occurring within less than three months following administration of the microfilaricide ivermectin, which renders infection undetectable by skin snip. However, reduction in skin mf due to ivermectin would have reduced the symptoms given that mf are responsible for most onchocerciasis-related symptoms (14, 15). It is possible that some of the symptoms may not be onchocerciasis related, especially as control of onchocerciasis-related morbidity has been achieved in many places (2). Alternatively, symptoms could be onchocerciasis symptoms remembered from before ivermectin administration. Limitations surrounding sensitivity of skin snip microscopy test (16) means that continued presence sub-detectible mf loads cannot be ruled out with 100% certainty.

We reiterate the need for a less invasive and more sensitive test to detect active onchocerciasis infection. Such diagnostic is needed when high mf intensity would be suppressed in the skin and symptoms are unlikely to be encountered but transmission ongoing with vector surviving even better when low load of mf is ingested (17). Such a test is also necessary for point of care management of onchocerciasis when mass drug administration (MDA) will stop. In instances of treatment naïve individuals or settings (such as hypo-endemic places - not previously considered for treatment), use of the more acceptable Ov-16 Rapid Diagnostic Test (RDT) should be considered (18). Sensitization materials should be reviewed, considering current epidemiology of onchocerciasis alongside beneficiary perspective. In line with this, a holistic approach for skin health should be considered where possible.

Previous experience also influenced acceptability of TTd. People complained previous testing that they never received results or treatment from. This could have been related to our study, there were delays in intervention 1 between testing, results, and treatment which led to a low (71%) treatment start level. Equally, before this TTd intervention, the same area has been surveyed using skin snip which could be conflated in people's memories due to similarity. Further compounding, instances of un-returned results led people to hold negative assumptions about the test, including skin being used for magical rituals. This contributed to refusal rates, especially during intervention round 1. Improvements made in the second round through reinforced sensitisation, immediate result return, demonstration of live mf and immediate start of treatment improved the situation.

Once tested, doxycycline treatment was well accepted, reflected in high treatment start (83%) and completion (98%) levels of intervention 2. The lower start rate (71%) in intervention 1 resulted from delays in starting treatment and were thought to be due to semi-nomads and farmers relocating. During treatment, side effects were low throughout the study as participant received a meal with the doxycycline tablet. Along with the intended reduction in side effects, meal support enhanced acceptability of treatment by acting as an incentive to attend for medicines. Eating and taking treatment in the morning fit well with beneficiaries' activities and even enhanced their daily activity. Contrarily, CDDs stated that treatment put more burden on them as they often forwent their daily activities. Treatment options such as weekly approach for the semi-nomadic population or increase CDD compensation package should be considered.

TTd could influence the acceptability of community-directed treatment with ivermectin (CDTi) as there were concerns that the largely popular CDTi would be replaced by TTd. Some respondents mentioned that ivermectin is better than TTd, driven by beneficiary perspectives of health improvement after taking ivermectin. This view corresponded to ivermectin's rapid reduction of skin mf which alleviates symptoms (19). Some people stated that doxycycline did not improve their health. This aligned with findings that some people tested and treated in intervention 1 were still found infected in round 2, which may be due to doxycycline's longer time to alleviate symptoms by impacting mf production, since mf can stay in the skin for up to 12 months (20, 21) but intervention 2 occurred before the next MDA. This supports the need of combining TTd with ivermectin (22). Implementing CDTi immediately after skin snip collection followed by doxycycline treatment to people with detected mf, would give beneficiaries immediate and sustained symptoms clearance (23). It will also be more impactful by breaking the transmission cycle quicker as evident in the impact evaluation of these interventions and elsewhere (8, 24, 25). In addition, combining ivermectin and TTd will be cost effective by combining logistics, ensuring normal CDTi continues seamlessly during and after TTd intervention. Doubt was raised whether adding doxycycline treatment will make any change to the benefit of consistent ivermectin treatment. Ivermectin drug fatigue and changing community priorities are becoming apparent [illegible]any places in sub-Saharan Africa (26-28) and any strategy such as TTd that build on the CDTi platform are likely to inherit challenges. There is need to fully understand these challenges even if medicine is changed.

We conclude that TTd, achieved a significant participation level (57%) for a curative intervention. Though lower than the 65% recommended ivermectin coverage (2, 29), it impact

is felt in shorter term (8). However, we appreciate that the remaining 43% is still significant especially if distribution of mf infection is skewed toward these non-participants. It is acceptable by the beneficiaries, TTd contributing to the significant and rapid reduction in mf (8). We highlight the value TTd offered and recommend its consideration in future endeavours, with the following recommendations.

Recommendation 1: Planning and preparation. Planning should begin by exploring the community to identify potential participation issues including systematic refusal, census underestimates, minority, and frequently excluded groups.

Recommendation 2: Improving test sensitivity and adopting a holistic approach. Research to find more sensitive tests should continue and be used when available. In addition, TTd approach should incorporate general skin consultation to address participant's self-diagnosis of onchocerciasis based on general symptoms of skin diseases but testing negative.

Recommendation 3: Combine CDTi and TTd, optimising their timing. While implementing TTd, maintain CDTi simultaneously, testing and offering ivermectin treatment the same day along with starting doxycycline treatment immediately once the result is available. A follow-up TTd round could be added.

Recommendation 4: Update and adapt sensitization. Review, update and adapt sensitization methods, channels and content considering participants' needs. Include showing participants microfilaria under the microscope and ensure all messages are relevant from beneficiary perspectives and relatable by different population subgroups.

Recommendation 5: CDD motivation and compensation. Method of medicine delivery should be considered for modification to reduce burden on volunteers dispensing the medicine. Alternatively, improve compensation for CDDs as TTd is more time demanding than CDTi.

Partner acknowledgement

We remain indebted to NTD programme in Cameroon at National, Regional and District levels and the communities who supported all the activities. We are thankful to the Centre for Research on Filariasis and other Tropical Diseases (CRFilMT) for collecting and analysing samples.

We equally thank Alexandre Chailloux for producing the map showing the various communities, and Susan D'Souza and Louise Hamill for reviewing the manuscript.

References

1. Coffeng LE, Fobi G, Ozoh G, Bissek AC, Nlatté BO, Enyong P, et al. Concurrence of dermatological and ophthalmological morbidity in onchocerciasis. Transactions of The Royal Society of Tropical Medicine and Hygiene. 2012;106(4):243-51.
2. WHO. Ending the neglect to attain the sustainable development goals: a road map for neglected tropical diseases 2021–2030. World Health Organization; 2020.
3. WHO. Report of the consultative meetings on strategic options and alternative treatment strategies for accelerating onchocerciasis elimination in Africa. Geneva, Switzerland: World Health Organization. 2015.
4. Boussinesq M, Fobi G, Kuesel AC. Alternative treatment strategies to accelerate the elimination of onchocerciasis. International Health. 2018;10(suppl_1):i40-i8.
5. Senyonjo L, Oye J, Bakajika D, Biholong B, Tekle A, Boakye D, et al. Factors Associated with Ivermectin Non-Compliance and Its Potential Role in Sustaining Onchocerca volvulus Transmission in the West Region of Cameroon. PLOS Neglected Tropical Diseases. 2016;10(8):e0004905.
6. Nkot PB, Matthews G. Larvicide application to control Simulium damnosum (Blackfly) in the Sanaga River, Cameroon. International pest control. 2007;49:178-9.
7. WHO. Guide for decision making and implementation of vector control as alternative treatment strategies for elimination of onchocerciasis. African Programme for Onchocerciasis Control; 2015.
8. Kareen Atekem RD, Aude Wilhelm, Benjamin Biholong, Joseph Oye, Hugues Nana Djeunga, Philippe Nwane, Franklin Ayisi, Daniel Boakye, Joseph Kamgno, Elena Schmidt, Rogers Nditanchou, Laura Senyonjo. Evaluating an Alternative Treatment Strategy in accelerating Onchocerciasis in area of persistent transmission in West Region of Cameroon (in press).
9. Wanji S, Tendongfor N, Nji T, Esum M, Che JN, Nkwescheu A, et al. Community-directed delivery of doxycycline for the treatment of onchocerciasis in areas of co-endemicity with loiasis in Cameroon. Parasites & Vectors. 2009;2(1):39.
10. Trotignon G, Dixon R, Atekem K, Senyonjo L, Kamgno J, Biholong B, et al. Cost of implementing Doxycycline test and treat strategy for Onchocerciasis in Cameroon, among settled and semi-nomadic groups of Massangam (in press).
11. Bakajika D, Senyonjo L, Enyong P, Oye J, Biholong B, Elhassan E, et al. On-going transmission of human onchocerciasis in the Massangam health district in the West Region of Cameroon: Better understanding transmission dynamics to inform changes in programmatic interventions. PLOS Neglected Tropical Diseases. 2018;12(11):e0006904.
12. Katabarwa MN, Eyamba A, Nwane P, Enyong P, Kamgno J, Kueté T, et al. Fifteen years of annual mass treatment of onchocerciasis with ivermectin have not interrupted transmission in the west region of cameroon. Journal of Parasitol Research. 2013;2013:420928-.
13. Okoro N, Nwali U, Oli A, Innocent O, Somadina O, Shedrack E. The Prevalence and Distribution of Human Onchocerciasis in Two Senatorial Districts in Ebonyi State, Nigeria. American Journal of Infectious Diseases and Microbiology. 2014;2:39-44.
14. Kirkwood B, Smith P, Marshall T, Prost A. Relationships between mortality, visual acuity and microfilarial load in the area of the Onchocerciasis Control Programme. Transactions of the Royal Society of Tropical Medicine and Hygiene. 1983;77(6):862-8.
15. Little M, Breitling L, Basáñez M, Alley E, Boatin B. Association between microfilarial load and excess mortality in onchocerciasis: an epidemiological study. The Lancet. 2004;363(9420):1514-21.
16. Thiele EA, Cama VA, Lakwo T, Mekasha S, Abanyie F, Sleshi M, et al. Detection of Onchocerca volvulus in Skin Snips by Microscopy and Real-Time Polymerase Chain Reaction: Implications for Monitoring and Evaluation Activities. The American journal of tropical medicine and hygiene. 2016;94(4):906-11.

17. Basáñez M, Townson H, Williams J, Frontado H, Villamizar N, Anderson R. Density-dependent processes in the transmission of human onchocerciasis: relationship between microfilarial intake and mortality of the simuliid vector. Parasitology. 1996;113(4):331-55.
18. Golden A, Faulx D, Kalnoky M, Stevens E, Yokobe L, Peck R, et al. Analysis of age-dependent trends in Ov16 IgG4 seroprevalence to onchocerciasis. Parasites & Vectors. 2016;9(1):338.
19. Opoku NO, Bakajika DK, Kanza EM, Howard H, Mambandu GL, Nyathirombo A, et al. Single dose moxidectin versus ivermectin for Onchocerca volvulus infection in Ghana, Liberia, and the Democratic Republic of the Congo: a randomised, controlled, double-blind phase 3 trial. The Lancet. 2018;392(10154):1207-16.
20. Turner JD, Tendongfor N, Esum M, Johnston KL, Langley RS, Ford L, et al. Macrofilaricidal activity after doxycycline only treatment of Onchocerca volvulus in an area of Loa loa co-endemicity: a randomized controlled trial. PLoS neglected tropical diseases. 2010;4(4):e660.
21. Hoerauf A, Specht S, Marfo-Debrekyei Y, Büttner M, Debrah AY, Mand S, et al. Efficacy of 5-week doxycycline treatment on adult Onchocerca volvulus. Parasitology research. 2009;104(2):437-47.
22. Turner JD, Tendongfor N, Esum M, Johnston KL, Langley RS, Ford L, et al. Macrofilaricidal Activity after Doxycycline Only Treatment of Onchocerca volvulus in an Area of Loa loa Co-Endemicity: A Randomized Controlled Trial. PLOS Neglected Tropical Diseases. 2010;4(4):e660.
23. Masud H, Qureshi TQ, Dukley M. Effects of Ivermectin with and without doxycycline on clinical symptoms of onchocerciasis. Journal of the College of Physicians and Surgeons--Pakistan. 2009;19(1):34-8.
24. Abegunde AT, Ahuja RM, Okafor NJ. Doxycycline plus ivermectin versus ivermectin alone for treatment of patients with onchocerciasis. Cochrane Database Syst Rev. 2016(1):CD011146-CD.
25. Hoerauf A, Mand S, Volkmann L, Büttner M, Marfo-Debrekyei Y, Taylor M, et al. Doxycycline in the treatment of human onchocerciasis: kinetics of Wolbachia endobacteria reduction and of inhibition of embryogenesis in female Onchocerca worms. Microbes and Infection. 2003;5(4):261-73.
26. Adekeye O, Ozano K, Dixon R, Elhassan EO, Lar L, Schmidt E, et al. Mass administration of medicines in changing contexts: Acceptability, adaptability and community directed approaches in Kaduna and Ogun States, Nigeria. PLOS Neglected Tropical Diseases. 2020;14(11):e0008857.
27. Dean L, Page S, Hawkins K, Stothard R, Thomson R, Wanji S, et al. Tailoring mass drug administration to context: implementation research is critical in achieving equitable progress in the control and elimination of helminth neglected tropical diseases in sub-Saharan Africa. International Health. 2016;8(4):233-4.
28. Chami GF, Kontoleon AA, Bulte E, Fenwick A, Kabatereine NB, Tukahebwa EM, et al. Community-directed mass drug administration is undermined by status seeking in friendship networks and inadequate trust in health advice networks. Social science & medicine (1982). 2017;183:37-47.
29. WHO. Community directed treatment with ivermectin: report of a multi-country study. World Health Organization; 1996.

Figure

Click here to access/download;Figure;ATS_TTd_qual_final_03092022_figur

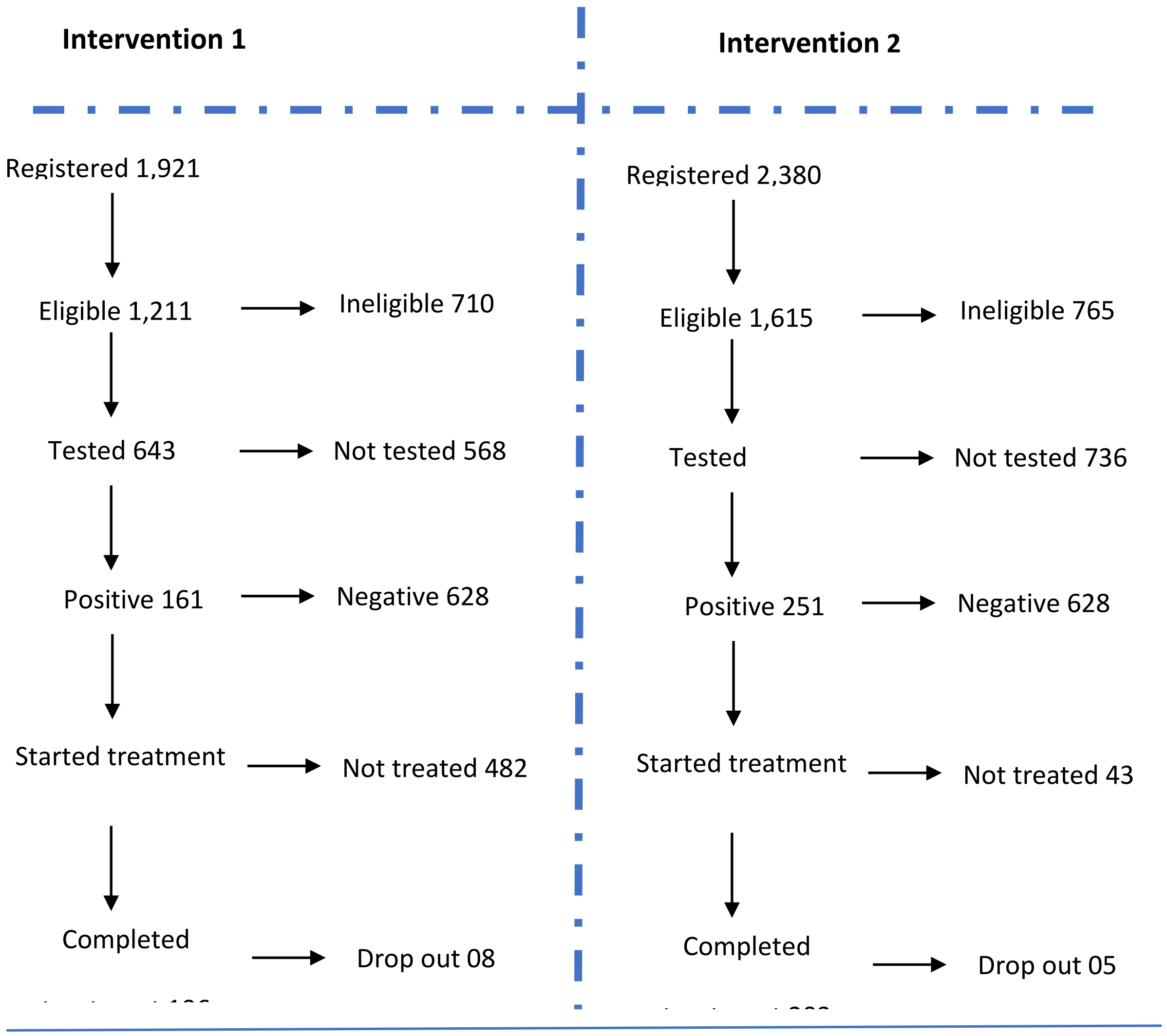

Figure 1. Flow chart from registration to treatment for interventions 1 and 2

Table
Click here to access/download;Table;ATS_TTd_qual_final_03092022_tables

Table 1. Summary of qualitative evaluation participants

Participants		KIIs (individuals)	#FGDs (group discussion)
Programme personnel		5	-
Community Drug Distributors	Semi-nomadic	0	-
	Settled	5	1
Community and opinion leaders	Semi-nomadic	2	-
	Settled	8	-
Community Members	Semi-nomadic	5	7
	Settled	5	9
Total		30	17

Table 2. Intervention 1 and 2 screen and treatment participation levels

Intervention 1									
**Categories (data from participants aged ≥9 yrs)**		**Registered**	**Test participation**		**Infection (positivity)**		**Treatment start**		**Course finalisation**
	**Sub-categories**	**n (%)**	**n (%)**	**Odds (P)**	**n (%)**	**Odds (P)**	**n (%)**	**Odds (P)**	**n (%)**
**Overall**		1,211	643 (54)		161(24)		114 (71)		106 (93)
**Sex (1,211)**	Females	610 (50)	317 (52)	ref	66 (21)	**ref**	37 (56)	**ref**	34 (92)
	Males	601 (50)	326 (54)	1.1 (0.428)	95 (29)	**1.6 (0.007)**	77 (80)	**3.4(0.001)**	72 (94)
**Age groups (1,211)**	9-15 yrs	291 (24)	147 (51)	ref	25 (17)	ref	19 (76)	ref	17 (89)
	16-25 yrs	329 (27)	155 (47)	0.9 (0.398)	41 (26)	**1.8 (0.036)**	19 (46)	**0.3 (0.021)**	17 (89)
	26-40 yrs	308 (25)	173 (56)	1.3 (0.166)	52 (30)	**2.2 (0.005)**	38 (73)	0.9 (0.784)	36 (95)
	>40 yrs	283 (23)	168 (59)	**1.4 (0.033)**	43 (26)	1.7 (0.051)	38 (88)	2.4 (0.190)	36 (95)
**Communities (1,211)**	Makouopsap	427 (35)	262 (60)	ref	45 (18)	ref	30 (67)	ref	27 (90)
	Makakoun	504 (42)	310 (61)	1.0 (0.823)	103(34)	**2.3 (<0.001)**	74 (72)	1.3 (0.527)	69 (93)
	Njinja/Njingouet	280 (23)	102 (35)	**0.3 (<0.001)**	13 (14)	0.8 (0.564)	10 (77)	1.7 (0.484)	10 (100)
**Community stay (1,211)**	< 2 yrs	114 (9)	50 (44)	ref	3 (6)	ref	1 (33)	ref	1 (100)
	2-5 yrs	149 (12)	96 (64)	**2.3 (0.001)**	14 (15)	2.9 (0.106)	9 (64)	3.6 (0.341)	9 (100)
	6-10 yrs	216 (18)	120 (56)	**1.6 (0.044)**	27 (23)	**4.8 (0.014)**	15 (56)	2.5 (0.476)	14 (93)
	> 10 yrs	763 (63)	377 (52)	1.4 (0.130)	117 (31)	**7.1 (0.001)**	89 (76)	6.4 (0.571)	82 (92)
**Intervention 2**									
**Categories (data from participants aged ≥9 yrs)**		**Registered**	**Test participation**		**Infection (positivity)**		**Treatment start**		**Course finalisation**
	**Sub-categories**	**n (%)**	**n (%)**	**Odds (P)**	**n (%)**	**Odds (P)**	**n (%)**	**Odds (P)**	**n (%)**
**Overall**		1,615	879 (54)		251 (29)		208 (83)		203 (98)
**Sex (1,615)**	Females	795 (49)	410 (52)	ref	96 (24)	ref	67 (70)	ref	63 (94)
	Males	820 (51)	469 (59)	1.3 **(0.023)**	155 (33)	**1.6 (0.002)**	141 (91)	**4.4 (<0.001)**	140 (99)
**Age group (1,615)**	9-15yrs	432 (27)	213 (49)	ref	30 (14)	ref	24 (80)	ref	21 (88)
	16-25yrs	388 (24)	177 (46)	**0.9** (0.291)	49 (27)	**2.4 (0.001)**	33 (67)	0.5 (0.227)	33 (100)
	26-40yrs	453 (28)	258 (57)	**1.4 (0.023)**	82 (32)	**2.9 (<0.001)**	68 (83)	1.2 (0.720)	68 (100)
	>40yrs	342 (21)	231 (68)	**2.1 (<0.001)**	90 (39)	**3.9 (<0.001)**	83 (92)	3.0 (0.071)	81 (98)
**Community (1,615)**	Makouopsap	535 (33)	310 (58)	ref	108 (33)	ref	92 (85)	ref	88 (96)
	Makakoun	678 (42)	332 (49)	**0.7 (0.002)**	106 (32)	0.9 (0.449)	85 (80)	0.7 (0.351)	84 (99)
	Njinja/Njingouet	402 (25)	237 (59)	1.0 (0.756)	37 (16)	**0.3 (<0.001)**	31 (84)	0.9 (0.838)	31 (100)
**Community stay (1,615)**	< 2 yrs	65 (4)	41 (63)	ref	4 (10)	ref	4(100)	--	4 (100)
	2-5 yrs	417 (26)	187 (45)	**0.5 (0.007)**	33 (18)	2.0 (0.222)	29 (88)	1.4 (0.514)	28 (97)
	6-10 yrs	231 (14)	121 (52)	0.7 (0.127)	34 (28)	**3.6 (0.023)**	25 (74)	0.6 (0.179)	24 (96)
	> 10 yrs	904 (56)	530 (59)	0.8 (0.495)	180 (34)	**4.8 (0.003)**	150 (83)	1.0 (--)	147 (98)
**Tribes (1025)**	Bamoun	494 (48)	298 (60)	ref	119 (40)	ref	96 (81)	ref	93 (97)
	Semi-nomads	212 (21)	102 (48)	**0.6 (0.003)**	7 (7)	**0.1 (<0.001)**	7 (100)	--	6 (85)
	Others	319 (31)	174 (55)	0.8 (0.103)	74 (43)	1.1 (0.531)	63 (85)	1.4 (0.430)	63 (100)

Table 3. Intervention 1 and 2 test, infection and treatment rates comparison within categories

Outcome	Categories	Subcategories	Intervention 1			Intervention 2		
			**Adjusted OR**	**p-value**	**Fitness of Regression Model**	**Adjusted OR**	**p-value**	**Fitness of Regression Model**
**Test rate**	**Community**	Makouopsap	Ref	--	LR χ2= 79.53; p<0.001	Ref	--	LR χ2= 106.62, p< 0.001
		Makakoun	1.0	0.901		**0.9**	**<0.001**	
		Njinja/Njingouet	**0.3**	**0.001**		1.89	0.086	
	**Age group**	9-15yrs	Ref	--		Ref	--	
		16-25yrs	0.9	0.348		1.1	0.728	
		26-40yrs	1.2	0.207		**1.5**	**0.024**	
		>40yrs	1.4	0.062		**2.3**	**<0.001**	
	**Sex**	Females	Ref	--		Ref	--	
		Males	1.0	0.934		1.1	0.394	
	**Tribes***	Bamoun	---	---		Ref	--	
		Semi-omads (Bororo)	---	---		**0.4**	**<0.001**	
		Others	---	---		0.9	0.576	
**Infection rate**	**Community**	Makouopsap	Ref	--	LR χ2= 41.82; p<0.001	Ref	--	LR χ2= 82.36, p< 0.001
		Makakoun	**2.5**	**<0.001**		**0.6**	**0.014**	
		Njinja/Njingouet	0.9	0.662		0.5	<0.180	
	**Age group**	9-15yrs	Ref	--		Ref	--	
		16-25yrs	**2.2**	**0.007**		**2.3**	**0.006**	
		26-40yrs	**2.5**	**0.002**		**1.8**	**<0.038**	
		>40yrs	1.7	0.06		**3.0**	**<0.001**	
	**Sex**	Females	Ref	--		Ref	--	
		Males	**1.7**	**0.004**		**1.6**	**0.020**	
	**Tribes***	Bamoun	---	---		Ref	--	
		Semi-nomads (Bororo)	---	---		**0.1**	**<0.001**	
		Others	---	---		1.1	0.556	
**Treatment rate**	**Community**	Makouopsap	Ref	--	LR χ2= 31.39; p<0.001	Ref	--	LR χ2= 34.57, p< 0.001
		Makakoun	0.9	0.779		0.6	0.257	
		Njinja/Njingouet	2	0.388		0.7	0.464	
	**Age group**	9-15yrs	Ref	--		Ref	--	
		16-25yrs	0.3	0.039		0.6	0.415	
		26-40yrs	1.1	0.899		1.3	0.689	
		>40yrs	3.1	0.105		**4.1**	**<0.029**	
	**Sex**	Females	Ref	--		Ref	--	
		Males	**3.7**	**0.001**		**5.1**	**<0.001**	

*Variable tribe including semi-nomad (Bororo) category was included only in round 2 following field observation that there were minority semi-nomadic population present and their inclusion appear challenging. There was no significant difference within the tribe categories regarding treatment start level (table 2), thus this is not included in the multiple logistic regression modelling with treatment (completion) as outcome.

Click here to access/download
Preprint PDF
ATS_TTd_qual_final_03092022.pdf
