# Supplementary material for: Acceptability of test and treat with doxycycline against Onchocerciasis in an area of persistent transmission in Massangam Health District, Cameroon
Source: PLoS Negl Trop Dis. 2023 Apr 5;17(4):e0011185. doi: 10.1371/journal.pntd.0011185 (PMC10075443; doi:10.1371/journal.pntd.0011185)
Supplement: Attachment — Submitted filename: Response Letter _PNTD-D-22-01137.docx [file pntd.0011185.s009.docx]

**PNTD-D-22-01137, Acceptability and effectiveness of test and treat with doxycycline against Onchocerciasis in an area of persistent transmission in Massangam Health District, Cameroon**

**Responses to reviewers’ comments**

Dear Editor,

We thank you and the reviewers for your precious time spent to review this manuscript and providing valuable feedbacks and insightful comments. We have carefully considered the comments and address to the best of our ability every one of them. We hope the manuscript after revision meets the standard. The responses to the comments raised are detailed below. They are arranged in a comment-response manner, with line references from the version with track changes. Will have include a version with changes highlighted as track and a clean version

 We welcome any further constructive comments.

Sincerely,

*RN*

Rogers NDITANCHOU

**Comments from the Reviewers**

**<b>Methods</b></br></br>
-Are the objectives of the study clearly articulated with a clear testable hypothesis stated?</br>
-Is the study design appropriate to address the stated objectives?</br>
-Is the population clearly described and appropriate for the hypothesis being tested?</br>
-Is the sample size sufficient to ensure adequate power to address the hypothesis being tested?</br>
-Were correct statistical analysis used to support conclusions?</br>
-Are there concerns about ethical or regulatory requirements being met?</br></br>**

**Reviewer #1:**

**Comment:** The authors should describe in detail what regression models were used, and what statistical analysis was conducted.

**Response:** Thank you for your feedback.

We have provided details of the regression model, lines: 209-220.

**Comment:** The authors described those surveys and discussions took place through group discussions and Key Informant Interviews. The method used to include participants in these groups, the method used to conceive the content of these survey and the discussions should be described. Furthermore, the whole content of these tools should be included in the manuscript (forms questions, answers of the participants, sensitization content, etc...)

**Response:** The detail of method FGD and KII have been included, lines 231-271.

The questionnaire and Excel sheet with all quotes from the transcribed tape records are included as supplementary materials (see S7 and S8_text.pdf); lines 875-877

**Comment:** The authors have also described quantitative and qualitative surveys, field notes, and meeting reports which lead to adjustments in the implementation. All these elements should be described in the method section.

**Response:** Thank you. We referred to skin snip test as quantitative survey. Field notes and meeting minutes/reports have been included. Lines 221-230

**Comment:** Other important missing information concerns the description of the skin snip method, how patients were informed, how the census was conducted, how adverse event where recorded, and the origin of the drug (doxycycline).

**Response:** Thank you. These details have been included:
Skin snip methods lines 166-180; how patients were informed, lines 178-180; how adverse event were recorded, lines 188-193; Origin of doxycycline, intervention 1(Vibramycin, Pfizer in the 1st round; Generic Doxycycline from the National Drug Store in 2nd round) lines 186-188.

**Comment:** Lines 94-96 This sentence is not clear; it is suggested to change it as follows: This intervention was supported by field monitoring, detailed notes, and a mid-point qualitative evaluation. Please clarify what it is meant by ‘mid-point qualitative evaluation’

**Response:** Thank you. The sentence has been reworded and clarification given for mid-point qualitative evaluation, lines 122-126.

**Comment:** Lines 96-97 It is suggested to change it as follows: In addition, partners on the field provided reports and meeting minutes summarizing encountered challenges and adjustments made during the implementation. Please provide further details about these challenges and adjustments or better, please provide a summary in the supplementary material. Otherwise, consider removing this sentence.

**Response:** Thank you. The sentence has been adjusted: lines 123-125. Additionally, meeting reports and changes effected are provided in the supplementary materials S2-4, lines 868-870

**Comment:** Line 98 Could you please elaborate on what was done to mobilize and sensitize the communities? Could you also elaborate on the census?

**Response:** Thank you this has been elaborated: Mobilisation and sensitisation: lines 128-146); census: 147-156.

**Comment:** Line 99 Are the eligibility criterion only ‘above 8 years of age, ‘non pregnant nor breastfeeding’, and ‘without severe illnesses?

**Response:** Thank you. It includes all: This is corrected Lines: 159-160

**“(**above the age of 8, not pregnant, not breast feeding and not having severe illness)”

**Comment:** Lines 108-110 This sentence is not very clear, it is suggested to modify it as follows: ‘A logistic regression model was used to compare the screening participation, the positivity proportion, the treatment starts and the proportion of participant completing the treatment course in both interventions. This analysis was refined by considering subgroups such as sex, age, community, length of stay in the community, and tribe’. Please provide additional information on the type of regression models used, the parameters, and the assumptions used, and lastly summarize the identified trends. This information could be provided as supplementary material or in the result section.

**Response:** This has been adjusted: 210-221; the trend identified is summarize in lines: 334-352

**Comment:** Lines 110-112 As above, it seems that the authors want to put emphasis on a process that was put in place to monitor the field intervention. Perhaps, instead of mentioning that meetings and minutes have taken place, it would be useful to describe when meetings were organized, what is their scope and what were the outcomes. Concretely, it would be useful to understand what field observations were made, and which adjustments were made.

**Response:** Thank you. We have included in lines 122-127; 222-231. Further details of field observation and adjustment are provided in supplementary materials as field notes and meeting minutes (see S2-4), lines 866-868.

**Comment:** Lines 118-131 The authors should explain what the focus group discussion and the Key Informant Interviews are. It would be also useful to understand in detail how these groups are formed, how the discussion is structured, and what questions were asked. The link with table 1 is also not clear. A legend for table 1 should be provided.
**Response:** Thank you. The focus group discussion and the Key Informant Interviews and how groups were formed are elaborated in lines 232-271. Furthermore, FGD and KII guides with questions and prompt are provided in supplementary material – S6, lines 873-875; the link with the table is provide in lines: 244-245.; a legend has been included: 253-255.

**Reviewer #2:**

**Comment:** The objectives of the manuscript are clearly stated, including the intention to describe the findings from field operations, what has been used and the consequent utilization of quality-quantitative methods to assess the intervention acceptance, uptake and barriers by the target population.

**Response:** Thank you.

**Reviewer #3:**

**Comment:** Are the objectives of the study clearly articulated with a clear testable hypothesis stated?
could be more clear what the main objectives of this paper is - there is a companion paper in press which might also be reporting some overlapping data. The paper reference both 'effectiveness' ie the parasitological efficacy of implementing doxycycline in reducing skin mf and 'acceptability' ie adherence of the alternative strategy in the communities trialled. But there is no real analysis of the effectiveness. Is this because it is reported elsewhere? The authors need to clarify

**Response:** Thank you. We have published the whole package and impact of the Alternative Treatment Strategies (ATS):

Atekem K, Dixon R, Wilhelm A, Biholong B, Oye J, et al. (2022) Evaluating the impact of alternative intervention strategies in accelerating onchocerciasis elimination in an area of persistent transmission in the West Region of Cameroon. PLOS Neglected Tropical Diseases 16(12): e0010591. <https://doi.org/10.1371/journal.pntd.0010591>.

This paper detailed the TTd aspect. The two papers were co-written with this one making reference to the other and without overlapping report. Initially effectiveness was regarded in this paper as participation or coverage of the intervention (not impact). However, we have now removed effectiveness. We focus here on of TTD component in the communities trialled – particularly the doxycycline test and treat aspect, lines 108-112

**Comment:** -Is the study design appropriate to address the stated objectives?
yes mixed methods quant and qualitative methods to capture 'acceptability' varying between different community groups.
**Response:** Thank you

**Comment:** -Is the population clearly described and appropriate for the hypothesis being tested?
Need more details in the flow chart to match all the various % calculations discussed in the results which I found very confusing and hard to follow.
**Response:** Thank you. The percentages have been added.

**Comment:** I am also really not clear how this intervention is separated geopgraphically from other alternative strategies implemented (twice annual CDTi, vector control) which are mentioned in the text. Much more clarity and information needs to be provided to determine whether there is a risk that overlapping alternative strategies are not influencing either 'effectiveness' or 'acceptability' of TTd?

**Response**: Thank you. The whole ATS package and its impact is reported in the sister paper to which this is linked:

Atekem K, Dixon R, Wilhelm A, Biholong B, Oye J, et al. (2022) Evaluating the impact of alternative intervention strategies in accelerating onchocerciasis elimination in an area of persistent transmission in the West Region of Cameroon. PLOS Neglected Tropical Diseases 16(12): e0010591. <https://doi.org/10.1371/journal.pntd.0010591>.

We focus here on 'acceptability' ie adherence to doxycycline test and treat aspect of the alternative strategies in the communities trialled – particularly the, lines 108-112. How interventions are were implemented are included in lines: 102-108; and in figure 1.

**Comment:** -Is the sample size sufficient to ensure adequate power to address the hypothesis being tested?

a generally moderate to large sample size for the multivariate logistic regression The sample size for FGD and KII were not justified
**Response:** Thank you. We have included that the sample size was reached first by self-selection based on the criteria of participants and community organisation; and then analysing to ensure saturation.

Lines: 233-245; 266-268

**Comment:** -Were correct statistical analysis used to support conclusions?
yes although the summary statistics were very confusing and attention needs to be paid to the flow chart to clarify this.

**Response:** Thank you. We have adjusted this in the figure and text, lines 334-352.

**Comment:** Two important analyses I found were missing:
1. change in mf prevalence baseline to follow up ie 'effectiveness' (no statistics - is this because this is being reported elsewhere?)

**Response:** Thank you. The whole ATS package and its impact is reported in the sister paper to which this is linked:

Atekem K, Dixon R, Wilhelm A, Biholong B, Oye J, et al. (2022) Evaluating the impact of alternative intervention strategies in accelerating onchocerciasis elimination in an area of persistent transmission in the West Region of Cameroon. PLOS Neglected Tropical Diseases 16(12): e0010591. <https://doi.org/10.1371/journal.pntd.0010591>.

We focus here on 'acceptability' ie adherence to doxycycline test and treat aspect of the alternative strategies in the communities trialled – particularly the TTd, lines 108-112.

**Comment:** 2. analysis that round two participant enrolment was SIGNIFICANTLY higher than round one.

**Response:** Thank you. We have included this in lines 290-306

**Comment:** -Are there concerns about ethical or regulatory requirements being met?
YES - it was not clear whether pregnancy test was done or the requirement for this in ethics. This is standard for Test and Treat with a class D drug.

**Response:** Thank you. Pregnancy test was done. We have now elaborated on this:

Line 161-163

--------------------

**<b>Results</b></br></br>
-Does the analysis presented match the analysis plan?</br>
-Are the results clearly and completely presented?</br>
-Are the figures (Tables, Images) of sufficient quality for clarity?</br></br>**

**Reviewer #1:**

**Comment:** The result section should be improved:
Figure 1 needs to be corrected (see below). A legend describing tables 2 and 3 should be added, in addition, a detailed explanation should be included in the text.

**Response:** Thanks this has been corrected as follows:

Figure 1 (now figure 2) correction lines 6-20; legend describing table 2 – lines 327-333; legend describing table 3 – lines 365-371.

**Comment:** As in the method section, qualitative and quantitative assessments are described, however, there is no description of these assessments nor details on how they have been analyzed.
As commented in the method section, the regression models and the statistical analysis are not adequately described. It is essential in this section to clearly explain what statistical test and what regression model was used to analyze the data and how this analysis is liked with the conclusions.

**Response:** Thank you. This has been addressed.

Qualitative analysis lines 272-278. Quantitative analysis & regression model, lines 210-221; the trend identified is summarize in lines: 334-352.

**Comment:** Figure 1
Please provide a legend summarizing the eligibility criterion. Please clarify why residents were not tested or treated. Please define the dropout. Please check the numbers: there is an inconsistency in line 9, a missing number in line 8, and line 20 the dropout should be written as 8 and 9 (not 08 and 09), finally it seems that a line was deleted (line 21).

**Response**: Thank you. Figure 1 (now figure 2), legend has been provided, lines 23-30; inconsistency corrected, line 9; missing number included line 10; and dropout number corrected , line 19. Missing number in Line 20 has been added. Dropout defined, line 23.

**Comment:** Line 143 Please clarify the number of the intervention. I guess it is intervention 1.

**Response:** Thank you. This is intervention 2. Line 288.

**Comment:** Line 144 Please clarify the eligibility criterion or refer to the method section.

**Response:** Thank you. This has been clarified. “>8 years, not pregnant, not breastfeeding, not severely ill”. Lines 288-290.

**Comment:** Line 145 what test? Skin snip?

**Response:** Thank you. It is skin snip test. This has been corrected. Line 299.

**Comment:** Line 150-151 Please explain the reason why not all patients were not tested. Why do the authors report different percentages of participation based on the total and the eligible population? It is not obvious why this is relevant.

**Response:** Thank you. The reasons for non-participation have been added: Lines 306-307.

Only percentage of eligible population is reported. Here, we consider total eligible population register in the two rounds and skin snip test participation during intervention 1 and 2 together. Clarity has been added, 296-298.

**Comment:** Line 156 Please explain what quantitative assessment and what test.

**Response:** Thank you. Qualitative (you meant?) refers to the FGD and KII; and test refers to the skin snip test, lines 372-373.

**Comment:** Line 164 as above, what test?

**Response:** Thank you. It is the skin snip test. This has been corrected, line 380.

**Comment:** Line s165-168 What multiple regression model was used? This should be detailed in the method section and summarized in the result section. In table 3 the authors need to explain what the column's odds mean. Is it the probability to participate in a test? And what test? How are the odds of positivity defined? Is it mf in the skin? Or in the eyes? Or both? Please clarify what it is meant by ref. Same for the treatment start, what do odds mean in this case? Please provide an extensive legend and an appropriate explanation in the text.

**Response:** Thank you. This is corrected as follows:

Details of multiple regression use, lines 210-221; summary of results in lines: 334-352; legend for table 3 has been added with clarification of OR, lines 327-333

**Comment:** Line 171 Please defined the qualitative assessment. How was it done? How was it elaborated?

**Response:** Thank you. This has been added, lines 388-389; how is was done: 232-278

**Comment:** Lines 186-188 Please describe what changes were made. What was the impact of these changes on the second intervention compared to the first?

**Response:** Thank you. We have included the changes in supplement S2-S4, lines 404-414.

**Comment:** Lines 195-196 Please defined the qualitative assessment.

**Response:** Thank you. This is FGD and KII. These have been included, lines 413-414

**Comment:** Lines 204-208 It is not described how the census was conducted. Please explain what statistical analysis was conducted. As above, please describe the qualitative.

**Response:** Thank you. Description of the census and statistical analysis have been included in the method section: Census, lines 147-156; statistical analysis lines 210-221; Qualitative assessment is through FGD and KII, lines 423-424.

**Comment:** Lines 299 What is the method to report side effects?

**Response:** Thank you. This was based on beneficiary report when asked and noted in the register. This is included in method section lines, 194-195, 515.

**Reviewer #2:** (No Response)

**Reviewer #3:**

**Comment:** -Does the analysis presented match the analysis plan?

yes for acceptability evaluations apart from some instances where quantitative significant differences are not then discussed or followed up via FGD / KII (e.g. inter-community variation in acceptability)

**Response:** Thank you. We have included this discussion, lines 598- 610

**Comment:** Whereas the parasitological reduction in prevalence from 35% to 12% is not presented at all and only mentioned in the abstract. Thus, I am only guessing that the former aspect is reported elsewhere? If so, the authors need to re-write the paper to indicate that is is only focussing on acceptability with the efficacy of TTd reported elsewhere etc.

**Response:** Thank you. The whole ATS package and its impact is reported in the sister paper to which this is linked:

Atekem K, Dixon R, Wilhelm A, Biholong B, Oye J, et al. (2022) Evaluating the impact of alternative intervention strategies in accelerating onchocerciasis elimination in an area of persistent transmission in the West Region of Cameroon. PLOS Neglected Tropical Diseases 16(12): e0010591. <https://doi.org/10.1371/journal.pntd.0010591>.

We focus here on 'acceptability' ie adherence to doxycycline test and treat aspect of the alternative strategies in the communities trialled – particularly the TTd, lines 108-112.

**Comment:** -Are the results clearly and completely presented?
The table is OK - I can follow this. But I would like the flow chart to reflect the various summary % discussed in the results because this is extremely hard to follow.

**Response:** Thank you. The percentages have been added in figure 1 (now figure 2).

**Comment:** -Are the figures (Tables, Images) of sufficient quality for clarity?
Yes.

**Response:** Thank you.
--------------------

**<b>Conclusions</b></br></br>
-Are the conclusions supported by the data presented?</br>
-Are the limitations of analysis clearly described?</br>
-Do the authors discuss how these data can be helpful to advance our understanding of the topic under study?</br>
-Is public health relevance addressed?</br></br>**

**Reviewer #1:**

**Comment:** The conclusion seems reasonable. However, the method and result sections should be improved and aligned to support these conclusions. Currently, the manuscript lacks coherence and it isn't easy to link all sections.

**Response:** We have adjusted as per comments.

**Reviewer #2:**

**Comment:** Conclusions and results well presented, including public health implications and programatic relevance for future interventions. One recommendation would be to clearly state that data record can be vulnerable to error as it entails data entry to excel sheets from paper, which is prone to human error.

**Response:** Thank you. This limitation is now included, lines 199 -202.

**Reviewer #3:**

**Comment:** -Are the conclusions supported by the data presented?
I found the conclusions that doxycycline should be implemented with ivermectin as part of CDTi missing the point that this is a curative treatment which would not need to be delivered annually!

**Response:** Thank you. We are recommending that as doxycycline may not affect mf already produced and as they can live up to 12 months, giving it together with ivermectin once will clear mf which may become residual sustaining symptoms. Another point of maintaining CDTi is to ensure normal programmatic CDTi until formal stoppage decision. And if doxycycline impact does not reduce transmission to below eliminate threshold, CDTI would not be perturbed. Also, ivermectin is given to people aged 5-8, an age group that is ineligible for doxycycline. We have included more clarification on this, Lines 726-730.

**Comment**: Other conclusions on general implementation strategy are logical and supported by data.

**Response:** Thank you.

-Are the limitations of analysis clearly described?

**Comment:** certain limitations are alluded to 'delays in doxycycline treatment after testing' are alluded to but not fully explained.

**Response:** Thank you. This was due delay in obtaining doxycycline and inadequate coordination between partners. We have included this, lines 57-58, 450,635-639, 647-649

**Comment:** Is this population affected by other alternative strategies? this confounding limitation is not discussed.

**Response:** Thank you. The whole ATS package and its impact is reported in the sister paper to which this is linked: Atekem K, Dixon R, Wilhelm A, Biholong B, Oye J, et al. (2022) Evaluating the impact of alternative intervention strategies in accelerating onchocerciasis elimination in an area of persistent transmission in the West Region of Cameroon. PLOS Neglected Tropical Diseases 16(12): e0010591. <https://doi.org/10.1371/journal.pntd.0010591>.

We focus here on 'acceptability' ie adherence to the alternative strategies in the communities trialled – particularly the doxycycline test and treat aspect, 108-112.

**Comment:** Is this area of ongoing transmission?
**Response:** Thank you. We have stated this, lines 98-101

**Comment:** I have already mentioned that parasitological analyses are missing and so are limitations around the drop in prevalence indicated in the abstract
**Response:** Thank you. This paper is linked to another where impact is reported. In addition, The whole ATS package and its impact is reported in the sister paper to which this is linked: Atekem K, Dixon R, Wilhelm A, Biholong B, Oye J, et al. (2022) Evaluating the impact of alternative intervention strategies in accelerating onchocerciasis elimination in an area of persistent transmission in the West Region of Cameroon. PLOS Neglected Tropical Diseases 16(12): e0010591. <https://doi.org/10.1371/journal.pntd.0010591>.

We focus here on 'acceptability' ie adherence to the alternative strategies in the communities trialled – particularly the doxycycline test and treat aspect, lines 108-112.

**Comment:** -Do the authors discuss how these data can be helpful to advance our understanding of the topic under study?
yes

**Response:** Thank you.

**Comment:** -Is public health relevance addressed?
yes

**Response:** Thank you.

--------------------
**<b>Editorial and Data Presentation Modifications?</b></br><br/>
Use this section for editorial suggestions as well as relatively minor modifications of existing data that would enhance clarity. If the only modifications needed are minor and/or editorial, you may wish to recommend “Minor Revision” or “Accept”.**

**Reviewer #1:** (No Response)

**Reviewer #2:** (No Response)

Reviewer #3:

**Comment:** I have commented that the results text discussing eligibility, testing and treatment in rounds 1 and 2 really are very confusing to follow. A re-drawing of the flow diagram would help support the text. If the text could be split into
census
enrollment
testing
treatment commencement / completion for both rounds 1 and 2 that would aid the thread of the results. This should be mirrored by the flow chart.

**Response:** Thank you. We have adjusted the flow chart in figure 1 (now figure 2) to include percentages and split the text, lines 128, 157, 165, 180, and 196. We have also removed duplicated reference and added the suggested ones. Lines 766-796.

Find below links to requested papers:

- Turner JD, Tendongfor N, Esum M, Johnston KL, Langley RS, Ford L, et al. Macrofilaricidal activity after doxycycline only treatment of Onchocerca volvulus in an area of Loa loa co-endemicity: a randomized controlled trial. PLoS neglected tropical diseases. 2010;4(4):e660. <https://journals.plos.org/plosntds/article?id=10.1371/journal.pntd.0000660> )
- Hoerauf A, Specht S, Marfo-Debrekyei Y, Büttner M, Debrah AY, Mand S, et al. Efficacy of 5 -week doxycycline treatment on adult Onchocerca volvulus. Parasitology research. 2009;104(2):437-22. (<https://academic.oup.com/cid/article/60/8/1199/2462581?login=false>)

**Comment:** I also think the authors need to emphasise whether parasitological data on skin mf prevalence are to be discussed elsewhere and focus on acceptability of this approach OR to fully included these analyses here. It is an impressive reduction in skin mf prevalence only very tersely mentioned in the abstract! Clearly the intervention has been of benefit.

**Response:** Thank you. The whole ATS package and its impact is reported in the sister paper to which this is linked: Atekem K, Dixon R, Wilhelm A, Biholong B, Oye J, et al. (2022) Evaluating the impact of alternative intervention strategies in accelerating onchocerciasis elimination in an area of persistent transmission in the West Region of Cameroon. PLOS Neglected Tropical Diseases 16(12): e0010591. <https://doi.org/10.1371/journal.pntd.0010591>.

We focus here on 'acceptability' ie adherence to the alternative strategies in the communities trialled – particularly the doxycycline test and treat aspect, lines 108-112.
--------------------

**<b>Summary and General Comments</b></br></br>
Use this section to provide overall comments, discuss strengths/weaknesses of the study, novelty, significance, general execution and scholarship. You may also include additional comments for the author, including concerns about dual publication, research ethics, or publication ethics. If requesting major revision, please articulate the new experiments that are needed.**

**Reviewer #1:**

**Comment:** The content of this work illustrates well the difficulties to implement a field intervention, the differences between the communities, and the importance to engage with them for a successful intervention. This topic certainly deserves to be published in a scientific journal. However, in its current state the method, the presentation of the results, and the general coherence of the manuscript is not sufficient.
**Response:** Thank you. We have adjusted as per pervious comments.

**Comment:** In addition, the authors should include, at least in the supplementary material, all the results of the survey, meetings notes, a summary of discussions, and the material used for the sensitization and to inform patients. This includes all the blank forms, field forms, and all the modifications that occurred.

**Response:** Thank you. We have included meeting notes and discussion summary, field forms in supplementary materials, S1-S8), lines 863-873

**Recommend:** The authors could also have elaborated more on the feasibility to generalize community-directed distributions of doxycycline, the side effects of doxycycline, the pro, and cons compare to community-directed distributions of ivermectin, and lastly the anticipated efficacy.
**Response:** Thank you. We have included this, lines 680-711

**Comment:** The authors have described some feedback given by the treated communities. It would be interesting to elaborate more on the suitability of community-directed distributions of doxycycline in some communities over others.
**Response:** Thank you. We have included this in lines 680-711

**Comment:** Additional comments:
The abstract is difficult to follow and needs significant reworking. It should be shortened, to summarize the objective of this work and the main conclusions.

**Response:** We have reworked this in lines with the comments.

**Comment:** The statement of the Financial Disclosure is quite surprising (This work is funded by Sightsavers. The funder had no role in study design, data collection, and analysis, decision to publish, or preparation of the manuscript) when we see that the first author of the manuscript is affiliated to the Sightsavers office. This contradiction must be clarified.

**Response:** Thank you. It will be changed to: “This work is funded by public donations to Sightsavers. Employees of Sightsavers played pivotal roles in study design, data collection and analysis, the decision to publish, and preparation of the manuscript. There was no conflict of interest between the source of funds for research and the findings”

**Comment:** Reviewer #2: The study is relevant as address a programatic approach to a major neglected condition affecting vulnerable population.

**Response:** Thank you.

**Comment:** The method is clear, the study population also clearly stated. Regarding the study population, it would be relevant to add some overall comments and a brief description of main features of the semi nomadic groups covered in the study. As there are clear programatic recommendations targeting this group because of need to design culturally appropriate messages, adapt strategies to sensitize with tailored community messages, I believe the readers would benefit from further information of the group composition that might support findings as well as recommendations for future interventions.

Response: Thank you. This is included. 246-251

**Reviewer #3:**

**Comment:** There are many issues precluding immediate publication
There is an issue as to what the extent of this manuscript is discussing 'effectiveness' v 'acceptability' and a potential for duplicate data being published in parallel elsewhere - the authors need to be much more transparent regarding this or wait until the in press manuscript is in the public domain.

**Response:** Thank you. The whole ATS package and its impact is reported in the sister paper to which this is linked: Atekem K, Dixon R, Wilhelm A, Biholong B, Oye J, et al. (2022) Evaluating the impact of alternative intervention strategies in accelerating onchocerciasis elimination in an area of persistent transmission in the West Region of Cameroon. PLOS Neglected Tropical Diseases 16(12): e0010591. <https://doi.org/10.1371/journal.pntd.0010591>.

We focus here on 'acceptability' ie adherence to the alternative strategies in the communities trialled – particularly the doxycycline test and treat aspect, lines 108-112.

**Comment:** I have raised an issue of ethics regarding what was done to ensure pregnant women were not offered doxycycline.
**Response:** Thank you. Urinary pregnancy test was conducted for all women age >15 years. Lines 161-163.

**Comment:** The mode of action of doxycycline, its curative activity and thus its distinctiveness compared with the microfilaricide, ivermectin, needs much more attention in the manuscript. This is a novel approach and so the implementation required is radically different.

**Response:** Thank you. We have revised as advised, lines 76-95.

**Comment:** I have provided an annotated manuscript to help the authors.

**Response:** Thank you very much. We have followed this.

**Comment:** Finally, I am aware that two senior African scientists were involved in conceptualisation and implementation of this TTd study (Didier Bakajika & Samuel Wanji) are not on the authorship list - this may be an oversight but needs justifiying. Perhaps they are acknowledged in the 'in press' article that I have not had access to.

**Response:** Thank you. This was an oversight. They have been included, lines 8 & 9.

**Comment:** Further I am aware that this protocol was developed by the COUNTDOWN Consortium / Liverpool School of Tropical Medicine and that SightSavers consulted with COUNTDOWN regarding implementing this protocol prior to publication of the COUNTDOWN TTd protocol in 2019 (ie before it was in the public domain). Therefore, it is proper that COUNTDOWN is appropriately recognised in the acknowledgements section for sharing this information and providing advice.

**Response:** Thank you. We have included COUNTDOWN and Liverpool School of Tropical Medicine acknowledgement, lines 746-750.

--------------------
