# Supplementary material for: Acceptability of test and treat with doxycycline against Onchocerciasis in an area of persistent transmission in Massangam Health District, Cameroon
Source: PLoS Negl Trop Dis. 2023 Apr 5;17(4):e0011185. doi: 10.1371/journal.pntd.0011185 (PMC10075443; doi:10.1371/journal.pntd.0011185)
Supplement: Attachment — Submitted filename: Response Letter _PNTD-D-22-01137_v2.docx [file pntd.0011185.s010.docx]

**PNTD-D-22-01137, Acceptability of test and treat with doxycycline against Onchocerciasis in an area of persistent transmission in Massangam Health District, Cameroon**

16 February 2023

**Responses to reviewers’ comments**

Dear Editor,

We thank you and the reviewers for your precious time spent to review this manuscript and providing valuable feedbacks and insightful comments. We have carefully considered the comments and address to the best of our ability every one of them. We hope the manuscript after revision meets the standard. The responses to the comments raised are detailed below. They are arranged in a comment-response manner, with line references from the version with track changes. Will have include a version with changes highlighted as track and a clean version

 We welcome any further constructive comments.

Sincerely,

*RN*

Rogers NDITANCHOU

**Responses to reviewers**

<b>Key Review Criteria Required for Acceptance?</b></br></br>
As you describe the new analyses required for acceptance, please consider the following:</br></br>

<b>Methods</b></br></br>
-Are the objectives of the study clearly articulated with a clear testable hypothesis stated?</br>
-Is the study design appropriate to address the stated objectives?</br>
-Is the population clearly described and appropriate for the hypothesis being tested?</br>
-Is the sample size sufficient to ensure adequate power to address the hypothesis being tested?</br>
-Were correct statistical analysis used to support conclusions?</br>
-Are there concerns about ethical or regulatory requirements being met?</br></br>

Reviewer #1: Congratulations to the authors for improving the manuscript and addressing comments from the reviewers. The method section is now clear and the statistical method is well described.

Reviewer #3: OK

**Response**. Thank you for your critical inputs that improve the manuscript

--------------------

<b>Results</b></br></br>
-Does the analysis presented match the analysis plan?</br>
-Are the results clearly and completely presented?</br>
-Are the figures (Tables, Images) of sufficient quality for clarity?</br></br>

Reviewer #1: Results are well presented

Reviewer #3: OK

--------------------

<b>Conclusions</b></br></br>
-Are the conclusions supported by the data presented?</br>
-Are the limitations of analysis clearly described?</br>
-Do the authors discuss how these data can be helpful to advance our understanding of the topic under study?</br>
-Is public health relevance addressed?</br></br>

Reviewer #1: The structure supports more clearly the conclusions.

Reviewer #3: OK

**Response**. Thank you for your critical inputs that improve the manuscript

--------------------

<b>Editorial and Data Presentation Modifications?</b></br><br/>
Use this section for editorial suggestions as well as relatively minor modifications of existing data that would enhance clarity. If the only modifications needed are minor and/or editorial, you may wish to recommend “Minor Revision” or “Accept”.

Reviewer #1: line 153: the web link is repeated twice

**Response**. Thank you. We have removed the repeat. Line 153.

line 706: I agree that alternative diagnostics tools should be developed for Onchocerciasis. And, in agreement with the WHO guideline, the authors have suggested the use of a serological diagnostic tool based on the OV16 antigen.
It was however recently shown in Cameroon (10.1371/journal.pntd.0010380) that parasitological (skin biopsy) and immunological (OV16 antigen) parameters are in moderate agreement. I would therefore suggest mentioning the development of new diagnostics tools in general instead of insisting on OV16. Alternatively, the authors could provide a few additional examples of possible diagnostic techniques: the Multiplex (10.1186/s13071-019-3824-x), LAMP, etc...

**Response**. Thank you for your critical inputs. We have expanded the discussion and included the references. Lines 706-709.

As there are several acronyms in this article, the authors may consider including a small glossary.

Reviewer #3: nonw

**Response**. Thank you. We have included a small glossary (attachment).
--------------------

<b>Summary and General Comments</b></br></br>
Use this section to provide overall comments, discuss strengths/weaknesses of the study, novelty, significance, general execution and scholarship. You may also include additional comments for the author, including concerns about dual publication, research ethics, or publication ethics. If requesting major revision, please articulate the new experiments that are needed.

Reviewer #1: Congratulations to the authors for this work.

Reviewer #3: the authors have addressed my major comments and restricted their data and conclusions to acceptability of implementing a combination of alternative strategies for the elimination of onchocerciasis in a persistant community focus in West Cameroon. The paper is now sufficiently clear for publication, in my opinion.

**Response**. Thank you. We are happy with this view.
